# The key roles of cancer stem cell-derived extracellular vesicles

**DOI:** 10.1038/s41392-021-00499-2

**Published:** 2021-03-08

**Authors:** Chaoyue Su, Jianye Zhang, Yosef Yarden, Liwu Fu

**Affiliations:** 1 State Key Laboratory of Oncology in South China; Collaborative Innovation Center for Cancer Medicine; Guangdong Esophageal Cancer Institute, Sun Yat-sen University Cancer Center, Guangzhou, People’s Republic of China; 2 Key Laboratory of Molecular Target & Clinical Pharmacology and the State Key Laboratory of Respiratory Disease, School of Pharmaceutical Sciences & the Fifth Affiliated Hospital, Guangzhou Medical University, Guangzhou, People’s Republic of China; 3 Department of Biological Regulation, Weizmann Institute of Science, Rehovot, Israel

**Keywords:** Cancer stem cells, Tumour heterogeneity

## Abstract

Cancer stem cells (CSCs), the subpopulation of cancer cells, have the capability of proliferation, self-renewal, and differentiation. The presence of CSCs is a key factor leading to tumor progression and metastasis. Extracellular vesicles (EVs) are nano-sized particles released by different kinds of cells and have the capacity to deliver certain cargoes, such as nucleic acids, proteins, and lipids, which have been recognized as a vital mediator in cell-to-cell communication. Recently, more and more studies have reported that EVs shed by CSCs make a significant contribution to tumor progression. CSCs-derived EVs are involved in tumor resistance, metastasis, angiogenesis, as well as the maintenance of stemness phenotype and tumor immunosuppression microenvironment. Here, we summarized the molecular mechanism by which CSCs-derived EVs in tumor progression. We believed that the fully understanding of the roles of CSCs-derived EVs in tumor development will definitely provide new ideas for CSCs-based therapeutic strategies.

## Introduction

Currently, cancer remains the most devastating disease on a global scale. Based on the data from GLOBOCAN 2018, there are nearly 18.1 million new cancer cases worldwide, including ~9.6 million cancer deaths.^1^ In recent years, great progress has been made in the molecular mechanism of tumorigenesis and development.^2,3^ However, it is undeniable that cancer recurrence, metastasis, and therapeutic resistance remain a major challenge in the current treatment of cancer.^4^ Accumulated researches have confirmed that most malignancies consist of multiple heterogeneous populations, that is, tumors are heterogeneous. Based on the current knowledge, it has been shown that the progression to therapy-resistant and metastatic disease is due to the presence of so-called CSCs.^5,6^ The concept of CSCs states that there are various cancer cells with different phenotypes in tumor tissue bulk, and a small number of cancer cells have the ability to continuously self-renewal and be able to seed new tumors.^7^ In most cases, traditional antitumor therapies frequently cause recurrent tumor diseases. One of the reasons is that these treatments only target a large number of non-CSCs, but do not eliminate a small number of CSCs.^8^ The mechanisms by which CSCs generate resistance to conventional therapies are complex, including drug efflux, DNA damage repair, dormancy, and anti-apoptosis.^9^ In addition, it is worth noting that the difference between CSCs and non-CSCs is most likely caused by the epithelial–mesenchymal transition (EMT) process. After the activation of the EMT process, cancer cells lose epithelial properties and instead acquire interstitial properties, which leads to their enhanced stem-like phenotype.^8^

Extracellular vesicles (EVs) are important mediators of cell–cell communication. Research to date strongly supports that EVs contribute to tumor growth, drug resistance, metastasis, and tumor immune microenvironment remodeling.^10–13^ EVs from parental cells can be internalized by recipient cells, and achieve epigenetic regulation of the target cell genome.^14^ Recent studies have shown that CSCs-derived EVs play a key role in mediating tumor resistance, metastasis, stemness, and remodeling the tumor immune microenvironment.^15,16^ In this review, we focus on how CSCs-derived EVs affect the biological characteristics of non-CSCs. We hope that this summary will enable people to better understand the mechanism of EVs secreted by CSCs in mediating tumor progression and metastasis.

## Cancer stem cells

### The concept and feature of cancer stem cells

**Fig. 1:**
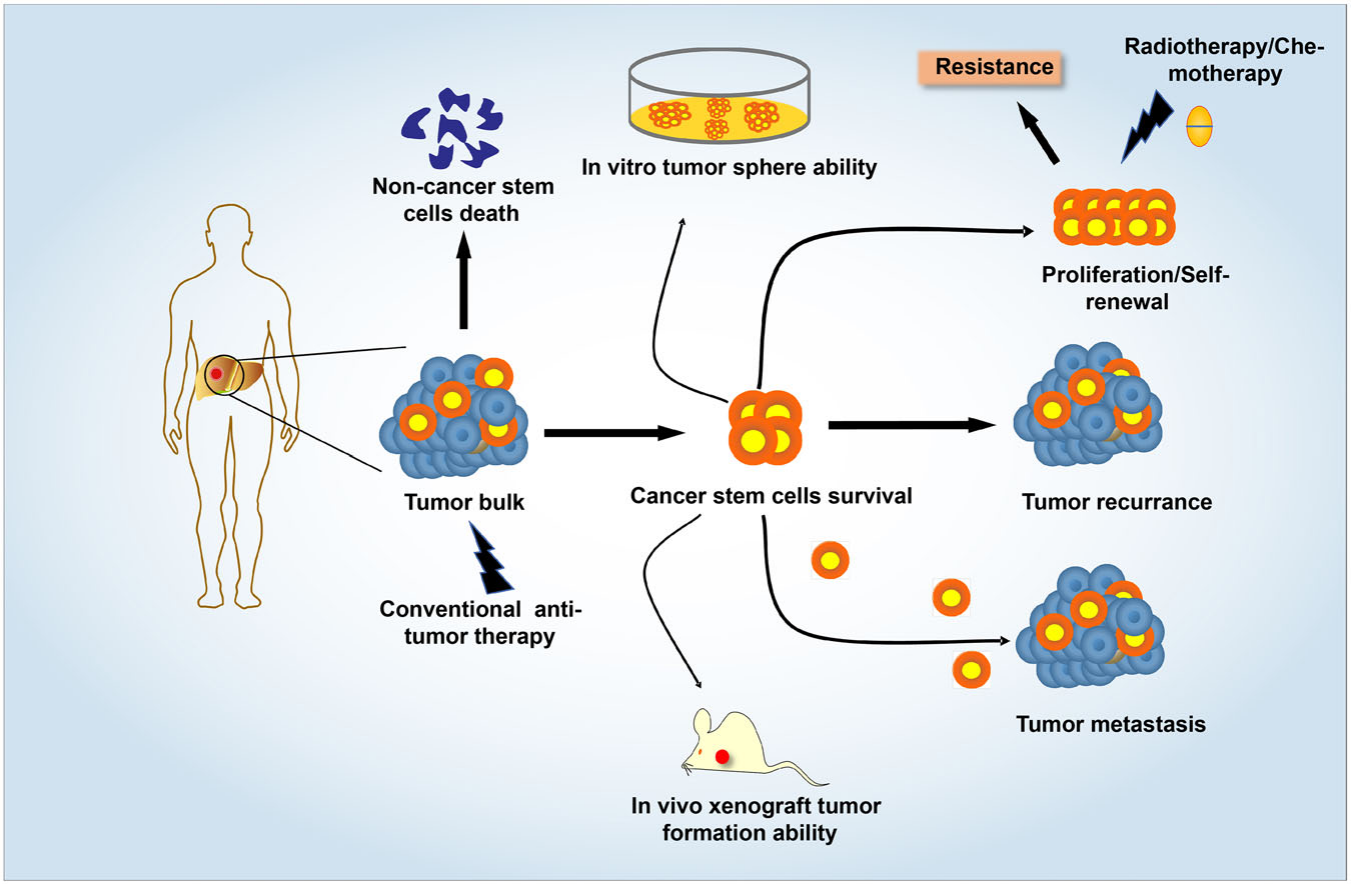
The characteristics of cancer stem cells. Due to the resistance of CSCs to conventional treatment, the majority of tumor patients have recurrent and metastatic disease after receiving conventional antitumor therapy. In vitro, a single CSC possesses the capability to form tumor spheroids, which represent the self-renew and proliferation ability of CSCs. In vivo, a small number of cancer stem-like cells can trigger tumor forming in mice. In addition, CSCs have inherent drug resistance and dormancy characteristics, as well as the ability to trigger distant metastasis of cancer

CSCs, also known as tumor-initiating cells, are the small population of cells in a tumor bulk, which represent a critical subset of the tumor population.^17,18^ The concept of CSCs was first proposed in the 1800s, and it was not until 1994 that Dick and colleagues successfully isolated leukemia stem cells for the first time, which strongly confirmed the theory of tumor heterogeneity.^17,19^ In subsequent studies, more and more researchers identified and isolated CSCs in solid tumors, and these isolated cells showed more tumorigenicity than non-CSCs in immunocompromised mice.^17,20^ The core of the concept of CSCs is the observation that not all cells in tumors are equal.^21^ That is, tumor growth is driven by a limited number of dedicated stem cells capable of self-renewal.^22,23^ Current researches show that CSCs resist radiation and chemical insults, and be able to stay dormant for a long time, as well as colonize in distant organ.^24,25^ A major attraction of the CSC concept rests in the explanations it provides for several well-known clinical phenomena: almost inevitable recurrence of tumors after initial successful chemotherapy and/or radiation treatment.^18,26^ In certain cancer patients, especially breast cancer patients, the metastasis of the primary tumor appeared many years after curative surgical treatment, which most likely due to quiescent CSCs that have metastasized to distant organs.^27^ An important feature of CSCs is their strong tumorigenicity in xenotransplantation in vivo. For example, a very small number of CD44^+^CD24^−^ (~100 cells) breast CSCs showed tumorigenicity in mouse xenotransplantation assays, whereas tens of thousands of cells with alternate phenotypes were not.^28^ After that, many researchers have conducted similar studies on other types of solid tumors, such as lung cancer,^29^ colon cancer,^30,31^ pancreatic cancer,^32^ prostate cancer,^33^ ovarian cancer,^34^ and brain cancer.^35^ In recent years, the focus of the CSC field has shifted to the use of freshly isolated tumor specimens and early-passage xenografts for transplantation research instead of using cultured tumor cells.^36^ Xenotransplantation assays have become an important means to assess CSCs subgroups and their activities.^18^ Based on the heterogeneity of the tumor, cell subgroups were sorted from the primary tumor and transplanted into immunodeficient mice by the limiting dilution method, after which tumor growth is scored some weeks or months later.^30,37^ The different tumor initiation abilities among tumor cell subpopulations can be explained as evidence for the presence of CSCs in the primary tumor^30^ (Fig. 1).

### Cancer stem cell model

It is now clear that tumors are heterogeneous, and that the heterogeneity of cancer arises from the genetic or epigenetic differences between the cancer cells themselves and diverse cell types initially recruited to the tumor.^38,39^ Two different cellular models have been proposed to explain tumor heterogeneity: the clonal evolution model and the CSC model.^38,40^ The clonal evolution model demonstrates that successive mutations accumulating in a given cell produce dominant clone populations, which thrive under the selection of microenvironmental pressure, and ultimately determine the tumor phenotype.^38,41^ However, the CSC theory postulates the hierarchical structure of cells. Only cells with the characteristics of stem cells or progenitor cells promote tumorigenesis and establish the inherent cellular heterogeneity of the primary tumor.^36,42^ It is generally believed that CSCs undergo symmetric division to replenish the CSC pool and irreversible asymmetric division to generate non-CSCs with low tumorigenic potential.^38,43^ However, the evolving evidence adds new insights to CSC theory. CSCs themselves do not exist as a static population. CSCs and non-CSCs have the potential to transform each other, and there are many different types of CSCs in a single tumor.^43,44^ Although the clonal evolution and CSC theory explain the heterogeneity of tumors and the occurrence and development of cancer from two different perspectives, in some cases, tumors show the characteristics of both models.^45^

### Cancer stem cell marker

At present, the gold standard for identifying CSCs includes in vitro tumorsphere formation and in vivo limiting-dilution tumorigenicity assays in immunocompromised mice.^46^ Nevertheless, studies have demonstrated that many cell surface markers can be used to isolate CSCs-rich subtypes in various types of solid tumors and hematological malignancy, including CD133, CD44, CD90, CD34, ALDH1, EpCAM, etc.^47–49^ Although these markers are not specifically expressed by CSCs, it is still feasible to achieve in vitro enrichment of CSCs subgroups through a combination of one or more markers.^50^ For example, the combination of CD34, CD38, and IL3Rα can achieve the prospective separation of leukemia stem cells.^17,51^ In addition, CD133, a pentaspan membrane glycoprotein, is one of the most well-characterized biomarkers used for the isolation of CSCs.^52^ CD133 was first used as a marker for glioblastoma stem cells (GSCs).^53^ In primary GSCs, only the CD133^+^ subpopulation, but not CD133^−^ cells, has the ability to maintain tumorigenesis and produce heterogeneity.^54^ In recent years, it has been found that CD133 and other CSCs markers (such as integrin α6 and ALDH) co-expression in tumor cells, and the combination of these markers improves the CSC phenotype.^55^ CD44 and ALDH1 are two other common CSCs surface markers.^56^ They can be used alone or in combination with other markers to isolate cancer cells with stemness characteristics. The combination of CD44^+^CD24^−^ and ALDH1^+^ has been widely used to isolate a variety of solid CSCs, especially for the enrichment of breast CSCs and oral squamous cell carcinoma stem cells.^57,58^ Moreover, recent studies have found many more powerful CSCs markers.^59^ For example, SSEA-1 (stage-specific embryonic antigen) was identified as a CSC marker in both human glioblastoma and syngeneic mouse models of medulloblastoma.^59^ In addition to identifying and enriching CSCs subgroups, these CSCs markers are also used to assist in cancer detection, prognosis assessment, and cancer diagnosis.^60^ Here, we summarized several common CSCs surface markers in a variety of solid tumors and hematological tumors (Table 1).

**Table 1:** Surface markers used for the identification of CSCs

Marker	Detected in healthy tissue	Expression in cancer stem cells	Refs.
CD133	Expressed in various cell types and tissue sites, especially proliferating cells	Breast, colon, brain, liver, lung, melanoma, ovarian, pancreatic, and prostate	^48,206,227^
CD44	Broadly on multiple tissues	Bladder, breast, colon, brain, gastric, head and neck, leukemia, liver, ovarian, pancreatic, and prostate	^48,206,228^
CD90	T cells; neurons	Breast, brain, liver, and lung	^48,206,229^
CD34	Hematopoietic and endothelial progenitors	Hematopoietic malignancies	^230^
CD24	Broadly on B cells; neuroblasts	Breast, colon, liver, ovarian, and pancreatic	^48,159^
CD38	Multiple stages of B and T cells	Negative on leukemia stem cell	^231^
CD71	Broadly on multiple tissues	Negative on gastric stem cell	^232^
CD15/SSEA-1	Myeloid cells; adult neural stem/progenitor cells	Brain and melanoma	^59,233^
CD54/ICAM1	Endothelial cells; pneumocytes; lymphoid cells	Gastric, liver, and esophageal	^232,234^
CD166/ALCAM	Membranous expression in various tissue	Colon, lung, melanoma, and prostate	^235–237^
CD177	Bone marrow, intestine, and lymphoid tissue	Lung, leukemia, and ovarian	^238,239^
ALDH1A1	Broadly on multiple tissues	Bladder, breast, colon, brain, gastric, head and neck, lung, pancreatic, and prostate	^48,190^
ABCG2	Broadly on multiple tissues	Brain, head and neck, lung, melanoma, osteosarcoma, and prostate	^240^
ABCB5	Keratinocyte progenitors	Melanoma	^241^
EpCAM	Pan-epithelial marker	Breast, colon, lung, and pancreatic	^206^
LGR5	Broadly on multiple tissues	Breast, colon, gastric, and head and neck	^48^
BMI-1	Broadly on multiple tissues	Breast, brain, head and neck, leukemia, pancreatic, and prostate	^242,243^
Integrin α6	Broadly on multiple tissues	Breast, prostate, and brain	^244,245^
CXCR4	Broadly on multiple tissues	Renal, breast, brain, and pancreatic	^48,246^
Nestin	Nerve cells; neural stem cell	Melanoma, brain, osteosarcoma, ovarian, and prostate	^247–249^

*SSEA-1* stage-specific embryonic surface antigen 1, *ICAM1* intercellular cell adhesion molecule-1, *ALCAM* activated leukocyte cell adhesion molecule, *ABCG2* ATP binding cassette subfamily G member 2, *ABCB5* ATP binding cassette subfamily B member 5, *EpCAM* epithelial cell adhesion molecule, *LGR5* leucine-rich repeat containing G-protein-coupled receptor 5, *CXCR*4 C–X–C motif chemokine receptor 4

### The biological function of cancer stem cells

It has been widely described that the existence of CSCs is an important driving factor leading to tumor recurrence and the development of drug resistance.^61,62^ The mechanisms by which CSCs are resistant to radiotherapy and chemotherapy are complex, including the upregulation of drug efflux pumps, enhanced DNA damage repair, and ROS elimination ability.^8,63,64^ Importantly, in recent years, many studies have confirmed that dormancy, the intrinsic property of CSCs, plays a key role in mediating tumor resistance.^65^ Liau et al.^66^ showed that GSCs evaded antiproliferative treatment by reversibly transformed into a slow-cycling state. In addition, quiescent bladder CSCs can be reactivated in response to chemotherapy-induced damage, which, in turn, repopulate residual tumors after treatment, similar to the role of normal stem cells in wound repair.^67^ Previous studies have shown that the activation of TGF-β signaling induces quiescent breast cancer cells.^68^ Consistently, the TGF-β-rich tumor microenvironment slows the proliferation of squamous cell carcinoma stem cells and confers resistance to cisplatin therapy.^69^ Taken together, these observations indicate that the persistence of dormant CSCs is a key factor leading to tumor resistance and recurrence.

In addition, another important biological function of CSCs is the potential for metastasis and colonization to distant organs.^70^ Tumor metastasis is a complex multistep process. Tumor cells need to pass through the basement membrane to enter the blood or lymphatic vessels.^71^ Next, circulating tumor cells (CTC) escape the surveillance of the immune system and extravasate from the blood, reach distant organs, and adapt to the new microenvironment, where they become metastasis-initiating cells (MIC).^71^ It is reported that MIC are evolved from CSCs.^71^ A study showed that in breast cancer patients, some of the CTCs that originated from the primary tumor showed a CSC phenotype.^72^ In addition, it is readily to detect tumorigenic CD44^+^CD24^−/low^ CSCs in pleural fluid and bone marrow in metastatic breast cancer.^28,73^ More importantly, many studies have shown that CSCs-mediated metastasis is closely related to the activated EMT state.^18^ For example, the overexpression of Snail, the master transcription factor of EMT in breast cancer cells, has shown enhanced tumor initiation and metastatic potential in mouse and human models.^74^ Gene expression profile analysis showed that EpCAM^+^ CD24^−^ CD44^+^ CSCs also expressed genes related to EMT.^75^

Previous studies have reported that CSCs exhibit special properties to avoid immune detection and eradication.^76^ Recently, a number of studies have shown that CSCs also generate an immunosuppressive, pro-tumorigenic immune milieu by regulating the activity of various immune cells.^76,77^ GSCs secrete a variety of cytokines and extracellular matrix components, such as periostin, colony-stimulating factor, TGF-β, and macrophage inhibitory cytokines, which drive the polarization of both tissue-resident macrophages and recruited macrophages toward an M2 phenotype.^78,79^ Consistently, ovarian CSCs also secrete cyclooxygenase-2 and CCL2 to promote M2 polarization of macrophages.^80^ In addition, CSCs express CD47 and interact with the macrophage receptor SIRPα to deliver phagocytic inhibition signals, resulting in the weakening of the anticancer activity of macrophages.^81,82^ CSCs are also able to inhibit proliferative T cell response and promote the expansion of pro-tumorigenic regulatory T (Treg) cells.^77^ The culture supernatant of a variety of solid CSCs has been shown to promote the proliferation of Treg cells in vitro, involving the secretion of a series of cytokines, such as TGF-β, IL-2, IL-8, and IL-10.^83–85^

## EVs classification, biogenesis, cargo, and functions

### EVs classification

EVs, ~30–2000 nm in diameter, contain a variety of biologically active molecules, such as nucleic acids, proteins, and lipids.^86,87^ The term “EVs” used in the literature generally refers to a variety of nanoscale membrane vesicles, including exosomes, microvesicles (MVs), and apoptotic bodies.^86,88^ The classification is based on their intracellular origin. Exosomes are small membrane vesicles of endocytic origin with a diameter of 30–150 nm, which have a lipid bilayer membrane structure.^89–91^ Differently, MVs diameter of ~200–2000 nm, produced by outward germination and fission of the donor cell plasma membrane.^87,92^ Nevertheless, there is increasing awareness of the size overlap between these two classes of EVs, especially in the smaller particle range.^93^ The diameter of the apoptotic bodies is ~500–2000 nm, which are relatively large vesicles formed in the process of apoptosis, containing the nucleus, proteins, and even organelles from the apoptotic cells^87^ (Table 2).

**Table 2:** Classification of EVs

EVs type	Size (nm)	Surface markers	Origin	Ref.
Exosomes	30–150	CD63, CD9, CD81	Endosomes	^94^
Microvesicles	200–2000	ARF6, VAMP3	Plasma membrane	^101^
Apoptotic bodies	500–2000	TSP, C3b	Plasma membrane	^101^

*ARF6* ADP ribosylation factor 6, *VAMP3* vesicle-associated membrane protein 3, TSP thrombospondin, C3b complement protein C3b

### EVs biogenesis

#### Exosome biogenesis

**Fig. 2:**
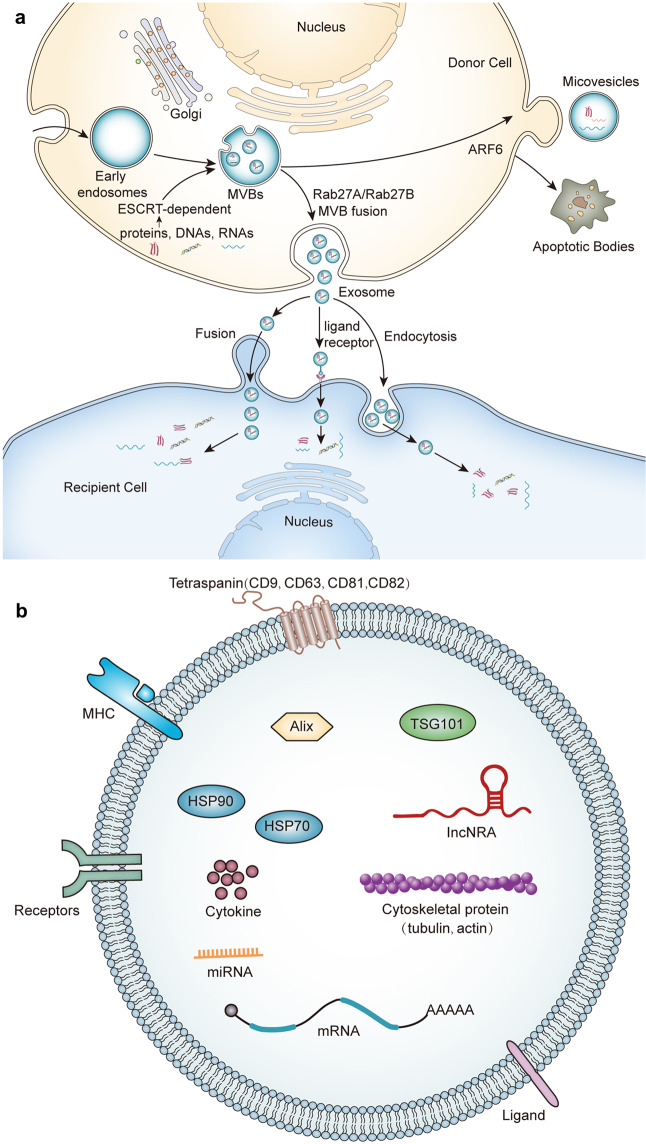
The classification, biogenesis, and content of EVs. **a** Exosomes originate from the reverse germination of the cell membrane. The cell membrane is recessed inward to form early endosomes, which are then sorted on the endoplasmic reticulum and processed on the Golgi apparatus to form multivesicular bodies. During this process, DNA, RNA, protein, and lipids in cells are sorted into vesicles mainly through ESCRT-dependent pathways. Under the regulation of the Rab family protein (Rab25/Rab27), MVBs fuses with the plasma membrane and are released into the extracellular space to form exosomes. Microvesicles are produced by outward germination and fission of the donor cell plasma membrane. GTP binding protein ARF6 of rho family members plays an important role in the formation of MVs. Few studies have reported the biogenesis of apoptotic bodies, which are currently considered to be relatively large vesicles derived from apoptotic cells. **b** EVs contain multiple types of cargoes, including nucleic acid, proteins, and liquids. EVs contain high levels of tetraspanins proteins (CD9, CD63, CD81, and CD82), MHC molecules, heat shock proteins (HSP 70 and HSP 90), and other transmembrane proteins and signal receptors

During the biogenesis of exosomes, endosomes are first formed by invagination of the plasma membrane, and then sorted on the endoplasmic reticulum and processed on the Golgi complex to form multivesicular bodies (MVBs).^93^ The vesicles contained in MVBs are also called intraluminal vesicles (ILVs), which are released into the extracellular compartment to form exosomes after the mature MVBs fuse with the plasma membrane.^89^ The four endosomal sorting complexes (ESCRT-0–III) required for transportation are the most widely described pathway for exosome biogenesis.^94^ DNA, RNA, and ubiquitinated proteins in cells are sorted into ILVs through ESCRT pathway.^94^ Among them, ESCRT-0 is responsible for the recruitment and internalization of proteins, while ESCRT-I and ESCRT-II are responsible for the formation of sprouts and promote the enzymatic deubiquitination of cargo proteins before the formation of ILVs.^95^ Finally, ESCRT-III is responsible for plasma membrane invagination and isolation to form MVB.^96^ In addition to ESCRT-dependent formation of exosomes, ESCRT-independent pathways involving neutral sphingomyelinase-dependent ceramide formation, as well as ADP ribosylation factor 6 (ARF6), and phospholipase D2 (PLD2), have also been reported.^97^ The fusion of MVBs with the plasma membrane, and thus exosome release, is regulated by several RAB GTPases (including RAS-related protein RAB7A, RAB11, RAB27A, RAB27B, and RAB35), as well as membrane fusion soluble N-ethylmaleimide-sensitive factor attachment protein receptor (SNARE) complex proteins^98^ (Fig. 2a).

#### Microvesicle biogenesis

In comparison with exosome biogenesis, much less is known about MVs formation.^93^ Unlike the biogenesis of exosomes, MVs release is directly budding through the plasma membrane without relying on exocytosis.^12^ It has been reported that the GTP binding protein ARF6 of rho family members plays an important role in the formation of MVs.^93,99^ ARF6-GTP-dependent activation of PLD initiates a signal cascade that promotes ERK recruitment and phosphorylation to the plasma membrane. Subsequently, phosphorylated ERK activates myosin light chain kinase (MLCK).^93^ MLCK-mediated MLC phosphorylation eventually leads to the release of MVs.^99^ In addition, RHOA-dependent rearrangement of the actin cytoskeleton is also an important process of plasma membrane germination to form MVs.^100^ The actin–myosin interaction shrinks the cytoskeleton structure and promotes the release of MVs^101^ (Fig. 2a).

### EVs cargo

In the process of EV biogenesis, EVs selectively enrich a series of cargo molecules with biological activity, including many types of RNA and proteins.^102,103^ Studies have shown that EVs encapsulate a large number of transport proteins, such as tubulin, actin and actin-binding molecules, as well as several proteins related to the specific functions of secretory cells.^91,104^ For exosomes, almost all exosomes carry MHC class I molecules and heat shock proteins (HSP), especially HSP 70, and HSP 90, which participate in antigen presentation and can bind antigen peptides to MHC class I molecules.^86^ In addition, exosomes also carry high concentrations of tetraspanins proteins (CD9, CD63, CD81, and CD82), signal receptors, and integrins, which are involved in antigen presentation, cell adhesion, immune regulation, and the pathophysiology of target cells.^105,106^ In addition to proteins, EVs also contain lipids, especially raft lipids, such as ceramides, sphingolipids, cholesterol, and glycolipid phospholipids.^107^ Importantly, EVs carry abundant microRNA, mRNA, lncRNA, and DNA, which can be transported to different cell types, thereby widely affecting the gene expression of target cells.^93^ Given that EVs selectively package many specific biomolecules from parental cells, they have broad application prospects in the development of cancer diagnostic markers and cancer tissue biopsies.^108^ In general, although much is known about the trafficking of cellular cargo molecules to EVs, our understanding of the underlying mechanism of cargo selection remains very much in its infancy^109^ (Fig. 2b).

### The biological functions of EVs

EVs are heterogeneous signal messengers secreted by cells, which can be recognized and absorbed by target cells to exchange membrane proteins and cytoplasmic contents between the two cell types and realize the transfer of cell epigenetic information.^105,110^ In terms of tumors, EVs mediate the communication between tumor cells and tumor-associated stromal cells, and tumor cells to promote tumor progression and metastasis.^111^ Tumor cells, together with tumor-associated stromal cells, release EVs to produce bidirectional cross talk.^112,113^ Importantly, the transmission of cancer EVs between tumor cell subgroups not only transfers the malignant phenotype, but also spread tumor heterogeneity.^91^ For example, studies have shown that EVs-mediated communication between different GSCs subpopulations leads to the generation of cancer cell subpopulations with intermediate phenotypes.^114,115^ Tumor cell-derived EVs activated VEGF signaling in endothelial cells, thereby promoting tumor angiogenesis.^116^ Interestingly, the components in EVs could change in response to the state of the parent cells.^117^ In a hypoxic environment, EVs secreted by cancer cells are rich in a variety of hypoxia-regulated RNA and proteins, which play an important role in inducing tumor angiogenesis and metastasis.^117,118^ EVs-mediated tumor cell metastasis has been widely reported. In recent years, more and more studies have focused on the role of EVs in the formation of tumor pre-metastatic niche.^119^ Tumor-derived EVs enter the blood circulation and reach distant organs, where they create a microenvironment that is conducive to tumor metastasis and colonization so that the scattered tumor cells can grow rapidly.^120^ Hoshino et al.^121^ showed exosomal integrins secreted by tumor cells are the decisive factor for tumor organotropic metastasis. Exosomal integrin α6β1, α6β4, and αvβ5 instigated lung fibroblasts, endothelial cells, and macrophages to differentiate into pro-tumor phenotype subtypes, thereby providing favorable soil for the colonization of CTCs.^121^

In addition, EVs also play a key role in mediating tumor drug resistance.^122,123^ Many studies have reported that the delivery of EVs secreted by drug-resistant tumor cells to sensitize tumor cells enhances the drug resistance of the latter.^13,122,124^ Drug-resistant tumor cells usually overexpress a variety of proteins related to drug efflux, including ABCG2, P-gp, ABCA3, and MRP1. These drug efflux pumps can be selectively sorted into EVs to transfer the resistance of parental cells.^125–128^ Moreover, a variety of miRNAs and lncRNAs in EVs also play an important role in promoting the development of tumor cell resistance.^129,130^ Overexpression of miR-221/222 was found in breast cancer drug-resistant cells and enriched in its EVs, and it was confirmed that its transfer to drug-sensitive cells would lead to the development of tamoxifen resistance.^131^ In HCC, lincRNA-VLDR and lincRNA-ROR in EVs have been proved to be key factors that mediate drug resistance of tumor cells.^132,133^ In addition, EVs secreted by a variety of tumor-associated stromal cells in the TME also promote the occurrence of tumor drug resistance.^134^ Studies have reported that mesenchymal stem cells (MSCs)-derived exosomes induce dormancy of breast cancer cells.^135^ Hu et al. showed that CAFs-derived exosomes enhanced the stemness and resistance of colorectal cancer cells.^136,137^ Therefore, EVs-mediated cross talk between the tumor and microenvironment is an important manner by which resistance can be transferred to sensitive cancer cells.

Another important biological function of EVs is to promote the generation of tumor immunosuppressive microenvironment.^113^ The immunosuppressive ability of tumor cell-derived EVs not only creates a good microenvironment for tumors, but also includes comprehensive changes to the overall immune system, making it easier for the tumor growth, and allowing the tumor to spread more aggressively.^138,139^ EVs cargo contains elements able to induce multiples immune cell dysfunction.^139^ EVs secreted by tumors carry inhibitory ligands, which negatively regulate the key receptors TCR and IL-2R on T cells, promote their activation and proliferation, as well as reprogram them to Th2 phenotype.^140^ In addition, Hsp72 on the surface of EVs induced IL-6/STAT3 signaling pathway through a TLR2-dependent mechanism, thereby activating myeloid suppressor cells.^141^ It has also been reported that cancer-derived EVs could promote monocytes to secrete a variety of pro-inflammatory cytokines, including IL-6, TNF-α, and IL-1β.^142^ Other immune cells are also regulated by cancer-derived EVs. For example, Treg cells respond to cancer-derived EVs to promote their proliferation and anti-apoptosis.^143^ Macrophages have the ability to polarize to M2 type macrophages after receiving cancer-secreted EVs.^144^

## The biological roles of CSCs-derived EVs

It is now clear that EVs derived from different types of cells are significantly different, both in the cargoes they carried and the functions they performed.^103^ As an important heterogeneous group in cancer tissues, CSCs secrete EVs that perform multiple biological functions, including promoting non-CSCs stem-like characteristics, chemotherapy resistance, metastasis, angiogenesis, and immunosuppression.^145,146^ Understanding the mechanism of cell communication in the TME mediated by such EVs is helpful for precision therapy targeting CSCs.^147^ Compared with non-CSCs-derived EVs, CSCs-derived EVs contain multiple stemness markers and proteins, such as CD133, CD44, Notch1, and the proteins in these EVs deliver to non-CSCs to enhance their stemness.^148–150^ Indeed, CSCs-derived EVs generate transient or dynamic tumor heterogeneity in the adjacent TME.^151^ CSCs secreted EVs carried specific proteins and transcription factors to neighboring cells has a greater impact on maintaining tumor heterogeneity.^152^

### CSCs-derived EVs promote non-CSCs to gain cancer stem-like phenotype

EVs actively participate in cell-to-cell interactions by shutting cellular components.^153^ There was strong evidence that CSCs-derived EVs promoted non-CSCs to acquire stem-like properties, leading to the enhanced tumorigenicity.^153–156^ Studies have found that EVs shed by CSCs carry the stemness markers of parent cells, which possess the ability to reprogram non-CSCs to obtain a stem-like phenotype.^149,157–159^ For example, CD44v6 and Tspan8, two markers of pancreatic cancer-initiating cells (PaCIC), have been detected in PaCIC-derived exosomes.^149,157^ Wang et al.^149^ showed that exosomes containing CD44v6 and Tspan8 derived from PaCIC promoted a shift toward stem cell features in CD44v6 knockdown and Tspan8 knockdown non-PaCIC. Exosomal CD44v6 and Tspan8 act as a hub, initiated by CD44v6-dependent RTK, GPCR, and integrin activation. In addition, it also affected miRNA processing in non-PaCIC.^149^ Therefore, a promising treatment for pancreatic cancer is to specifically block the interaction between PaCIC-exosomes and non-PaCIC, such as the use of RTK inhibitors to block signaling, and anti-Tspan8 to block exosome uptake.^149,157^ In addition to stemness markers-related proteins, studies have also found that CSCs-exosomes are wrapped with proteins related to activation of tumor stemness signaling pathways, which may directly activate the stemness-related signaling pathways on non-CSCs, thereby facilitating their stem-like phenotype.^155^ The enrichment of Notch1 protein was found in GSCs-derived exosomes.^155^ It is well known that the Notch signaling pathway in CSCs is abnormally activated, and Notch1 is a vital receptor on this signaling pathway.^160^ GSCs-derived exosomal Notch1 reprogramed non-GSCs to GSCs and significantly enhanced their tumorigenicity.^155^ After treated with Notch1 RNA interference or Notch inhibitors, GSCs-exosomes-treated non-GSCs showed reduced spheroid formation ability and stemness protein expressions.^155^

**Fig. 3:**
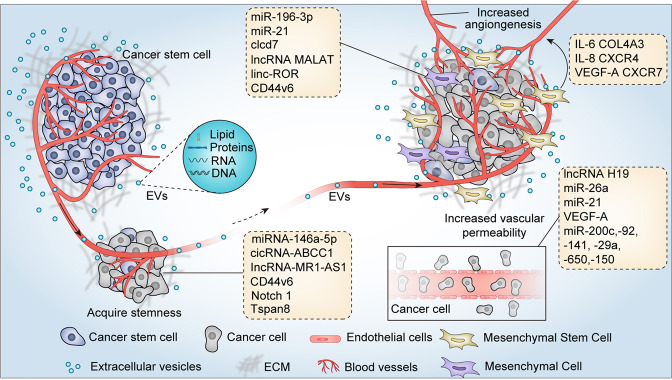
Cancer stem cells-derived EVs promote tumor metastasis, angiogenesis, and cancer stem-like phenotype. CSCs-derived EVs confer non-CSCs stem-like characterizes through delivering miRNA, lncRNA, cicRNA, and stemness-related proteins or activating stem-related signaling pathways. In addition, CSCs-derived EVs carry multiple bioactive molecules to promote tumor cells EMT, tumor angiogenesis, and vascular permeability, which make a significant contribution to cancer metastasis. CSCs-derived EVs also instigate mesenchymal stem cells to secrete a variety of signaling molecules, such as IL-6, IL-8, VEGF-A, COL4A3, CXCR4, and CXCR7, thereby promoting tumor angiogenesis

In addition, CSCs-derived EVs contain abundant RNA molecules that can reprogram non-CSCs to CSCs by activating certain stemness-related pathways.^153,154,160^ A study performed by Zhao et al. showed that exosomes secreted by CD133^+^ colorectal cells deliver circRNA-ABCC1 to non-colorectal CSCs, and promoted their stemness phenotype and sphere formation ability.^153^ Mechanistically, exosomal cicRNA-ABCC1 activated the Wnt/β-catenin pathway to promote the progression of colorectal cancer.^153^ Moreover, Li et al.^154^ showed that exosomal lncRNA FMR1-AS1 derived from ESCC stem cells transferred the stem-like characteristics to recipient non-CSCs in the TME. Exosomal lncRNA FMR1-AS1 bound to endosomal toll-like receptor 7 (TLR7) and activated downstream TLR7/NF-κB signaling to promote c-Myc expression, thereby inducing ESCC cell proliferation, anti-apoptosis, and invasion ability^154^ (Fig. 3).

### CSCs-derived EVs promote tumor metastasis

Metastatic tumors are responsible for >90% of cancer-related deaths.^71^ Recent studies showed that cancer cells with the ability to colonize in distant organs have the characteristics of CSCs.^161^ That is, metastatic cancer cells are the result of evolution and drive by CSCs. With the in-depth study of EVs, now we know that EVs are crucial for primary cancer metastasis, the formation of pre-metastasis niche, and the colonization of cancer cells at metastatic sites.^12^ Recent studies have investigated the roles of CSCs-derived EVs in tumor metastasis.^151,162^

A study performed by Wang and his colleagues showed that clear cell renal cell carcinoma (CCRCC) stem cells-derived exosomes accelerated the process of EMT and promoted lung metastasis.^151^ The study demonstrated that CCRCC stem cells-derived exosomal miR-19b-3p strongly promoted tumor cell EMT through targeting PTEN signaling pathway.^151^ More importantly, CD103 was enriched in CSCs-exosomes, which determined the organotropism metastasis of CCRCC to the lung, suggesting that CCRCC stem cells-derived exosomes possessed the ability to guide cancer cells to specific target organs, which was similar to the previous research results of Hoshino et al.^121,151^ Consistently, an increase in CD103^+^ exosomes was found in blood samples of CCRCC patients with lung metastasis.^151^ Similarly, another study also showed that the MVs shed by renal CSCs greatly enhanced the lung metastasis of renal cancer cells in mice.^163^ The researchers identified a group of miRNA expression profiles related to tumor prognosis and poor metastasis in renal CSCs-derived MVs, including miR-200c, -92, -141, -29a, -650, and -151.^163^ In addition, liver CSCs-derived exosomes significantly increased the invasion and metastasis of liver cancer cells (upregulation of P13K and ERK), and induced EMT (upregulation of TGFβ1).^164^ Interestingly, injection of bone marrow MSCs-derived exosomes could reverse this effect of liver CSCs-derived exosomes.^164^ In a study on lung cancer, the researchers showed that exosomes derived from lung CSCs transferred cargo miR-210-3p to non-lung CSCs, which significantly contributed to the pro-metastasis phenotype.^162^ Exosomal miR-210-3p upregulated the expression levels of N-cadherin, vimentin, MMP-9, and MMP-1, and downregulated E-cadherin expression in non-lung CSCs.^162^ Mechanically, exosomal miR-210-3p promoted cancer cell metastasis by targeting FGFRL1.^162^ Hardin et al.^165^ investigated the roles of thyroid cancer stem-like cell-derived exosomal lncRNA MALAT1 and linc-ROR in thyroid cancer metastasis. LncRNA MALAT1 and linc-ROR are expressed in multiple cancer types and are associated with cancer metastasis and EMT.^166^ It was found that lncRNA MALAT1 and linc-ROR, as well as the EMT marker SLUG and the stem cell transcription factor SOX2 in thyroid CSCs-derived exosomes, were significantly upregulated.^165^ Thyroid CSCs-derived exosomes induced EMT program of normal thyroid cells and increased their aggressiveness.^165^

In addition to packaging miRNA and lncRNA, studies also found that CSCs-derived EVs carried certain protein molecules, which play a vital role in mediating cancer metastasis.^149^ As mentioned earlier, pancreatic CSCs-derived exosomal CD44v6 reprogramed non-pancreatic CSCs, and enhanced their mobility and invasiveness.^149,157^ In addition, claudin7 (cld7), a cancer-initiating cell marker in gastrointestinal tumors, is closely related to tumor progression. Cld7^+^ CIC-derived exosomes selectively packaged cld7 molecules, which significantly restored the spread and metastasis of cancer cells when it transferred to cld7-knockdown tumor cells.^167,168^ RTK inhibitors can neutralize this effect caused by PaCIC-derived exosomes, suggesting that cld7 activates RTK signaling networks.^167^ Therefore, blocking RTK signaling pathway is a promising tool for interrupting PaCIC-exosomes activity (Fig. 3).

### CSCs-derived EVs promote tumor angiogenesis

On the one hand, CSCs drive angiogenesis mainly by releasing pro-angiogenic factors and exosomes. They can obtain blood to resist hypoxia in tumors by autophagy or directly forming tubular structures.^169^ On the other hand, the vascular niche in the TME also releases growth factors through adjacent and paracrine pathways to support the growth of CSCs and maintain its stemness.^170,171^ Recently, studies have reported that CSCs-derived EVs carried a variety of pro-angiogenic molecules, which promoted tumor angiogenesis through cross talk with endothelial cells and other stromal cells in the microenvironment.^172–174^

The selective packaging of specific miRNAs shed by CSCs-EVs has been demonstrated by further studies.^175^ For instance, a study based on GSCs showed that exosomes derived from GSCs with the gain-/loss-of-function of miR-26a significantly affect the angiogenesis of human brain microvascular endothelial cells.^172^ The overexpression of miR-26a in GSCs and their derived exosomes significantly enhanced tumor angiogenesis and increased the expression levels of VEGF, MMP-2, and MMP-9.^172^ Mechanistically, miR-26a activated the PI3K/Akt signaling pathway by targeting PTEN.^172^ Similarly, Sun et al.^174^ showed that GSCs-derived exosomes promoted the angiogenic ability of endothelial cells through the miR-21/VEGF/VEGFR2 signaling pathway. They purified CD133^+^ GSCs from the glioblastoma cell line U-251 and found highly enriched miR-21 and VEGF in the exosomes they secreted. Compared with the control group without GSCs-exosomes treatment, GSCs-exosomes co-incubated endothelial cells show stronger angiogenesis and higher VEGF expression.^174^ Studies have shown that the pro-angiogenic factor VEGF-A is enriched in CSCs-derived EVs.^173,176^ Recently, it was reported that glioblastoma stem-like cells-derived EVs enriched in VEGF-A.^173^ Treatment of brain endothelial cells with such EVs showed enhanced angiogenesis and vascular permeability.^173^ However, when treating GSCs with the VEGFR signaling inhibitors, imatinib and sunitinib, the EVs released from GSCs could not promote endothelial cell permeability.^173^ Spinelli et al.^177^ studied the unique gene expression profiles and stimulating activity on endothelial cells in different subtypes of GSCs-derived EVs. Proneural (PN) and mesenchymal (MES) are two subtypes of GSCs, and the EVs they produce have different marker profiles, proteomes, and endothelial-stimulating activities.^177^ Protein composition analysis revealed that there are 733 proteins were common for EVs from MES and PN GSCs, but 1036 and 154 were unique to these respective donors.^177^ Unlike other literature that focuses on the commonality of GSCs-EVs promoting angiogenesis, this study emphasizes their heterogeneity^177^ (Fig. 3).

Patients with a high proportion of CD105^+^ renal CSCs often show tumor metastatic disease.^178^ It was reported that MVs released by CD105^+^ renal CSCs enhanced tumor angiogenesis.^163^ Lindoso et al.^179^ demonstrated that renal CSCs-derived EVs recruited bone marrow MSCs and participated in tumor matrix remodeling. After ingestion of renal CSCs-derived EVs, the phenotype of MSCs changed significantly, including increased expression of genes related to matrix remodeling, angiogenesis, tumor growth, as well as cell migration.^179^ Tumorigenic MSCs in turn promoted angiogenesis and tumor growth in renal cancer.^179^ In addition, CSCs-like CD90^+^ liver cells regulated the endothelial phenotype by releasing exosomes containing lncRNA H19. The researchers found that exosomal lncRNA H19 of CD90^+^ liver CSCs significantly increased the expression of VEGF, and promoted heterotypic adhesion between endothelial cells and CD90^+^ liver CSCs.^16^ In a study on ovarian cancer, Vera et al.^180^ revealed the cross talk between small EVs released from ovarian cancer spheroids (OCS) and MSCs to exert tumor-promoting activity. Under the stimulation of cisplatin, small EVs derived from CSCs-rich OCS induced the migration of bone marrow MSCs and promoted their secretion of IL-6, IL-8, as well as VEGF-A.^180^ These cytokines secreted by bone marrow MSCs in turn stimulated the angiogenic activity of HUVEC cells, and thus contributing to tumorigenic processes.^180^ In summary, the above results indicate that EVs released by CSCs interact with tumor microenvironmental stromal cells to promote tumor malignant phenotype (Table 3).

**Table 3:** Functions of cargo in different types of cancer stem cells-derived EVs

Molecular type	Source	Downstream target	Functions	Ref.
miR-210	Pancreatic CSCs	mTOR	Gemcitabine-resistance; anti-apoptosis; inhibit cell cycle	^184^
miR-21-5p	Oral squamous cell carcinoma CSCs	PI3K/mTOR/STAT3	Cisplatin resistance	^185^
miR-155	Breast CSCs	TGF-β, FOXO3a, and C/EBP-β	Doxorubicin and paclitaxel resistance	^187^
miR-19b-3p	Renal CSCs	PTEN	Promote EMT and lung metastasis	^151^
—	Liver CSCs	P13K/ERK/ TGFβ1	Promote invasion, migration, and angiogenesis	^164^
miR-210-3p	Lung CSCs	FGFRL1	Promote EMT and metastasis	^162^
—	Renal CSCs	VEGF-A	Promote lung metastasis and angiogenesis	^179^
miR-26a	Glioblastoma stem cells	PTEN/PI3K/Akt	Promote angiogenesis	^172^
miR-21	Glioblastoma stem cells	VEGF/VEGFR2	Promote angiogenesis	^174^
miRNA-146a-5p	Colorectal CSCs	Numb	Promote tumor immunosuppression microenvironment	^198^
lncRNA MALAT1; linc-ROR	Thyroid CSCs	SLUG/SOX2	Promote EMT, invasion, and metastasis	^165^
lncRNA FMR1-AS1	Esophageal squamous cell carcinoma stem cells	TLR7/NF-κB/c-Myc	Promote cancer cell proliferation and stem-like phenotype	^154^
lncRNA H19	Liver CSCs	VEGF/ICAM1	Promote tube formation and cell–cell adhesion	^16^
lncRNA MALAT1	Glioblastoma stem cells	miR-129-5p/HMGB1	Promote inflammatory response	^195^
Claudin7	Gastric CSCs	RTK	Promote metastasis	^167^
VEGF-A	Glioblastoma stem cells	Not determined	Promote angiogenesis	^173^
CD44v6	Pancreatic CSCs	RTK/GPCR/integrin	Promote cancer stem-like phenotype and metastasis	^149^
circRNA-ABCC1	Colorectal CSCs	Wnt/β-catenin	Promote cancer stem cell-like phenotype and tumorigenicity	^153^
Notch1	Glioblastoma stem cells	Not determined	Promote cancer stem cell-like phenotype and tumorigenicity	^155^
Tenascin C	Brain tumor-initiating cells	Integrin α5β1/αvβ6; mTOR	Inhibit T cells proliferation and activation	^190^
—	Glioblastoma stem cells	STAT3	Promotes monocyte polarization to M2 macrophages and PD-L1 expression	^194^
Triphosphate RNAs	Colorectal CSCs	PRP/NF-κB	Promote the tumor phenotype of neutrophils and its survival	^189^

### CSCs-derived EVs transfer drug-resistant traits to non-CSCs

Drug resistance is an important feature of CSCs.^181,182^ Increasing evidence showed that EVs derived from CSCs carried a variety of biologically active molecules, such as miRNA and lncRNA.^183^ These EVs could be taken up by neighboring non-CSCs, which further activated certain drug resistance-related signaling pathways and enabled non-CSCs to acquire drug resistance phenotype. For example, in a study on pancreatic cancer, Yang et al.^184^ reported that exosomes derived from gemcitabine-resistant pancreatic CSCs confer resistance characteristics to gemcitabine-sensitive pancreatic cancer cells by delivering miR-210. Several resistance-related proteins have been found to be upregulated in sensitive pancreatic cancer cells, including MDR1, YB-1, and BCRP, which implied that pancreatic CSCs-derived exosomal miR-210 might mediate non-CSCs subsets resistance to gemcitabine treatment by increasing drug efflux.^184^ Functionally, miR-210 mediated the resistance of tumor cells to gemcitabine by activating the mTOR signaling pathway.^184^ In addition, oral squamous cell carcinoma (OSCC) stem cells-derived EVs contained miR-21-5p, which activated OSCC cells PI3K/mTOR/STAT3 signaling pathway, leading to the resistance of non-OSCC stem cells to cisplatin.^185^ Interestingly, colon cancer cells released CD133-containing MVs, which activated the KRAS signaling pathway of the recipient cells to increase cell proliferation and anti-EGFR drug resistance. In addition, the selective packaging and release of CD133 were regulated by RhoA-GTPase and Rac1-GTPase.^186^

Another key mechanism of tumor cell resistance to chemotherapy is the activation of the EMT program.^8^ Cells undergoing EMT can acquire CSCs-like features, exhibit a MES phenotype, and share key signaling pathways and drug resistance phenotypes with CSCs.^8^ it has been reported that exosomes secreted by breast CSCs promote the EMT phenotype of non-breast CSCs and confer resistance to them.^187^ The exosomal miR-155 downregulated the expression of c/EBP-β, TGF-β, and FOXO3a genes, resulting in the upregulation of EMT-related and stemness-related genes (BMI1, SLUG, SNAIL, SOX9, and EZH2) expression in breast-sensitive cells, and significantly increased resistance to doxorubicin and paclitaxel.^187^

### CSCs-derived EVs promote the formation of tumor immunosuppression microenvironment

Cell–cell interactions in the TME result in cancer progression.^188^ Tumors are highly heterogeneous tissues, and studies have reported the mechanism of interactions between CSCs and tumor-infiltrating immune cells.^188,189^ Brain tumor-initiating cells with cancer stem-like characteristics secreted exosomes containing tenascin C, which significantly inhibited the proliferation and activation of T lymphocytes.^190^ Exosomal tenascin C interacted with integrin α5β1 and αvβ6 on T cells, subsequently attenuated the expression of p-mTOR signaling.^190,191^ It was worth noting that another study reported that GSCs-derived exosomes did not directly interact with T cells to suppress T cell immune responses, but induced monocytic myeloid-derived suppressor cells and inhibited the maturation of monocytes.^192^ When CD14^+^ monocytes were removed from PBMC, the inhibitory effect of GSCs-exosomes on T cell proliferation could be partially rescued.^192^ In addition, the EVs released by CD105^+^ renal CSCs inhibited the maturation of dendritic cells and the immune response of T cells, which might be caused by HLA-G in EVs.^193^

Moreover, GSCs-derived exosomes transferred to monocytes trigger monocyte agonist protein reorganization, inducing the differentiation of monocytes into immunosuppressive M2 macrophages, accompanied by increased expression of PD-L1.^194^ Mechanism studies indicated that the upregulation of monocyte PD-L1 was mainly related to the increased expression of p-STAT3.^194^ Studies have shown that glioblastomas are infiltrated with a large number of microglial cells, which interact with glioblastomas and induce tumor immunosuppression.^195^ Recently, a study performed by Yang and his colleagues demonstrated that EVs lncRNA MALAT1 released from GSCs mediated LPS-induced inflammatory response of microglia by targeting miR-129-5p/HMGB1 (high mobility group box-1) protein alix.^195^ When co-incubating with GSCs-derived EVs, IL-6, IL-8, and TNF-α secreted by LPS-stimulated microglial increased significantly. Studies confirmed that both IL-6 and IL-8 promote angiogenesis in glioblastomas, while TNF-α induced glioma cell invasion.^196,197^ Inhibiting the release of EVs from GSCs might be a promising method for treating gliomas.

**Fig. 4:**
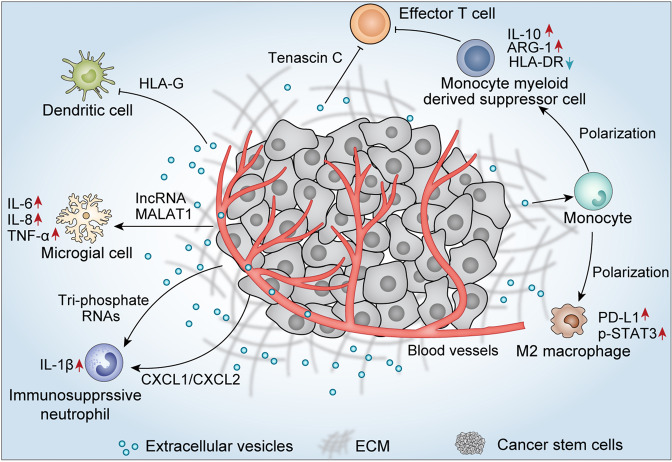
Cancer stem cells-derived EVs promote the formation of tumor immunosuppression microenvironment. EVs secreted by CSCs exhibited a tumor immunosuppression microenvironment through inhibiting the survival and proliferation of effector T cells and dendritic cells, as well as inducing the production of M2 macrophage, immunosuppressive monocytes, and neutrophils

Hwang et al.^189^ showed that colorectal CSCs-derived exosomes were enriched in mouse bone marrow, prolonged bone marrow neutrophil survival, and facilitated the tumor phenotype of neutrophils.^189^ Through a pattern recognition-NF-κB signaling axis, exosomal triphosphate RNAs promoted the increase of neutrophil IL-1β expression, thereby maintaining its own survival.^189^ In addition, colorectal CSCs also directly secrete CXCL1 and CXCL2 to recruit neutrophils to tumor tissues. Activated neutrophils secrete large amounts of IL-1β to promote tumorigenicity of colorectal cells.^189^ In addition, exosomal miRNA-146a-5p derived from colorectal CSCs promoted stemness and tumorigenicity by targeting Numb of colorectal cells.^198^ Patients with abundant exosomal miR-146a expression in serum exhibited higher CSCs traits and showed increased tumor-infiltrating CD66^+^ neutrophils, as well as decreased tumor-infiltrating CD8^+^ T cells, suggesting the production of an immunosuppressive microenvironment^198^ (Fig. 4).

## Differences between CSCs-derived EVs and non-CSCs-derived EVs in tumorigenesis

Although cancer cells and CSCs-derived EVs have many similar effects in promoting cancer progression, metastasis, and therapeutic resistance, there are still certain differences in their promotion of tumorigenesis due to the different cargo they package.^50,152^ Similar to non-CSCs-secreted EVs, CSCs-derived EVs also contain multiple RNAs, which performed specific biological functions different from non-CSCs-derived EVs.^152,199^ For example, a study performed by Wang et al.^151^ showed that exosomal miR-19b-3p derived from CCRCC stem cells initiated the EMT program of tumor cells and promoted metastasis. However, specific contributions of non-CSCs-derived exosomes miR-19b-3p in tumor progression have yet to be established.^192^ It is worth noting that a study reported that tubular epithelial cells-derived exosomal miR-19b-3p promoted the activation of M1 macrophage, driving the occurrence of tubular interstitial inflammation.^200^ Therefore, further research should consider the unique role of CSCs exosomal miR-19b-3p, which may be developed as a new target for the treatment of cancer. Similarly, exosomal miR-210-3p derived from lung CSCs contributed to the pro-metastatic niche of lung cancer, while in non-CSC, exosomal miR-210 mainly promoted tumor angiogenesis.^201^ In addition, colorectal CSCs-derived exosomes carried unique triphosphate RNAs to facilitate the formation of tumor immunosuppressive microenvironment.^189^ However, no studies have reported the role of triphosphate RNAs in EVs secreted by cancer cells. Non-CSCs and CSCs-derived EVs contain multiple lncRNAs, which also play different functions in promoting tumorigenesis, even if they are the same lncRNA.^165^ Exosomal linc-ROR derived from thyroid cancer stem-like cells showed the induction of normal thyroid cells EMT, and inculcate the local TME and the distant metastatic niche.^202^ Differently, in non-CSCs, such as hepatocellular cancer, EVs transferred linc-ROR was mainly related to the chemoresistance of cancer.^132^ In addition, CSCs-derived EVs may carry certain stemness-related signaling proteins, which could reprogram non-CSCs into CSCs.^203^ For example, GSC-released exosomes enhanced non-GSC stemness and tumorigenicity by transferring Notch1 protein.^148^ However, whether non-CSCs-derived exosomes could affect the biology phenotypes of CSCs has not yet been defined. Consistently, PaCIC secreted exosomes transferred CD44v6 protein (a biomarker of pancreatic CSC) to non-PaCIC and promoted their apoptosis-resistance, EMT, motility, and tumor progression.^149^ Collectively, the contents of CSCs-derived EVs have unique characteristics that are different from non-CSCs-derived EVs. However, in view of the current lack of research on CSCs-derived EVs, more basic research is needed for further demonstration.

## The cross talk between EVs and CSCs niche

Many CSCs rely on a specific set of external interactions with their microenvironment. CSCs niche comprises malignant cells together with inflammatory cells, vascular endothelial cells, fibroblasts, vasculature, and related matrix.^36,204,205^ The relationship between the CSC and the local environment appears to be bidirectional: the niche alters the cellular fate of cancer cells and, conversely, CSCs modify their microenvironment.^48^ Recent studies have shown that CSCs-derived EVs may become an important part in inducing tumor angiogenesis and vascular permeability.^173,175^ Indeed, CSCs in glioblastoma have been demonstrated to secrete VEGF that directly supports the development of the local vasculature.^172^ Treps et al.^173^ showed glioblastoma stem-like cells secreted VEGF-A in EVs, which significantly contributed to the in vitro elevation of permeability and angiogenic potential in human brain endothelial cells. Moreover, studies have also shown that CSCs-derived exosomes promote the transformation of monocytes into M2 macrophages, thereby mediating the formation of a tumor immunosuppressive microenvironment.^194^ Several kinds of research have reported that GSCs-derived exosomes could inhibit T cell activation, proliferation, and Th1 cytokine production, but did not affect the activation of Treg cells.^190,192^ In addition, primary cancer cells are also an important member of CSCs niche.^206^ Wang et al.^162^ demonstrated that exosomes derived from lung CSCs targeted non-CSCs fibroblast growth factor receptor-like 1 to promote the formation of pre-metastatic niche.

Interestingly, it has also been reported that EVs secreted by non-CSCs affect the stem-like phenotype of CSCs, as well as promote cancer drug resistance and metastasis.^9^ Shen et al.^207^ showed that the treatment with sublethal doses of chemotherapeutics induces breast cancer cells to secrete EVs with the ability to promote cancer stem cell-like phenotypes, rendering cancer cells resistant to therapy. In pancreatic ductal adenocarcinoma (PDAC), exosomal IncRNA Sox2ot promotes EMT and stem cell-like properties by regulating Sox2 expression.^208^ Kuc et al.^209^ reported that PDAC-derived exosomes promoted pancreatic CSCs motility. In addition, in prostate cancer, exosomes secreted by tumor cells under hypoxic conditions promoted the stemness and aggressiveness of naive prostate cancer cells.^210^ Taken together, these research demonstrated that CSCs-derived EVs, together with non-CSCs-derived EVs, make significant contributions to maintaining the CSCs niche.

## EVs-based therapeutic strategies for targeting CSCs

Given that CSCs are an important factor in tumor therapeutic resistance, there is an urgent need to find targeted therapies for this small subset of cells in tumor masses.^211–213^ Nanotechnology-based drug delivery systems are one of the most promising tools to achieve this goal in the clinic.^214^ EVs, particularly exosomes, as a natural nanovesicle have many advantages in acting as a drug delivery vehicle. Compared with synthesized nanoparticles, EVs have higher stability, biocompatibility and biodegradability, lower toxicity, and immunogenicity.^145,214,215^ Although there are still many challenges in using exosomes for the cancer treatment, many studies have developed exosomes-based nanocarrier drug delivery technologies.^216,217^ Exosomes can be engineered to have powerful targeting and delivery capabilities, and therefore showing great potential in CSCs targeted therapy.^145,218^ The development of EVs-based CSCs targeting technology will help improve tumor recurrence, drug resistance, and metastasis.^219,220^

Recently, many studies have achieved the therapeutic effect of targeting CSCs by constructing exosomes–nanoparticles as drug delivery vehicles. A study performed by Yong et al.^219^ developed a biocompatible tumor cell-secreted exosome-biomimetic porous silicon nanoparticles (PSiNPs), which can be used as a drug carrier for targeted cancer chemotherapy. When doxorubicin-loaded PSiNPs are ingested by tumor cells, they will be sorted and packaged into exosomes, and then secreted by tumor cells into the extracellular space.^219^ Exosomes-sheathed doxorubicin-loaded PSiNPs have the characteristic of being enriched in the side population cells with features of CSCs, resulting in the elimination of CSCs.^219^ In addition, Arabi et al.^221^ used anti-CD44 antibody-encapsulated liposomes to deliver doxorubicin to directly target CD44^+^ CSCs. Conceivably, anti-CD44 antibody-coated EVs could directly target CSCs and subsequently induce their death.^214^ Given that certain normal cells also express CSCs surface markers, it is possible to improve the efficiency of CSC targeting by using exosomes packaged with multiple antibodies. This is because normal cells may present one CSC surface marker, but rarely several of them simultaneously.^214^ Interestingly, Tian et al.^222^ engineered mouse immature dendritic cells to express a well-characterized exosomal membrane protein fused to αv integrin-specific iRGD peptide. The engineered exosomes exhibit potent targeting potential for αv integrin-positive breast cancer cells and significantly increased doxorubicin delivery efficiency in mice.^222^ Moreover, Qi et al.^223^ attached the superparamagnetic binding to transferrin on the surface of transferrin receptor-positive blood exosomes. Under the action of an external magnetic field, these exosomes are directed to the target tumor site to effectively inhibit the tumor growth. In addition, exosomes loaded with siRNA, miRNA, or small molecule inhibitors can be used as another method to achieve CSCs targeting.^224–226^ In summary, the success of these exosomes engineering methods will further improve the results of exosomes-mediated CSC targeting.

## Conclusion and perspective

The presence of CSC is a key factor in cancer recurrence, resistance, and metastasis. Conventional therapies usually eliminate a large number of non-CSCs population, but it is ineffective for CSCs population, leaving the possibility for the future development of local disease recurrence and/or metastasis. With the more extensive and in-depth research on EVs, people have gradually realized that EVs secreted by CSCs play a non-negligible role in tumor progression. For example, CSCs-derived EVs can reprogram sensitive tumor cells to have a drug-resistant phenotype like that of CSCs. Many studies have shown that the acquisition of cancer stem-like phenotypes is related to the EMT program, which indicates that CSCs are closely related to EMT. mRNA transcriptome sequencing revealed that EMT-related markers were significantly increased in the EVs of CSCs. Non-CSCs uptake of these EVs showed an activated EMT program. In addition, CSCs-derived EVs carry angiogenic active factors, such as VEGF, VEGF-A, which can significantly enhance the angiogenesis effect of endothelial cells. Therefore, it can be seen from the current discussion that many biological characteristics of cancer cells are determined by non-genetic mechanisms, and epigenetics also plays an important role in cancer progression.

However, there are still many challenges in the research and application of CSCs-EVs due to technical limitations and some practical problems. For example, currently, many studies on the separation of CSCs are based on surface markers, and this is not the most standard way. As we mentioned above, the gold standard for CSCs identification is still in vitro tumorsphere formation and in vivo limiting-dilution tumorigenicity assays in immunocompromised mice. In addition, it is difficult to ensure that all or most of the extracted EVs derived from CSCs subgroups. Moreover, it is difficult to harvest enough EVs from a small number of CSCs. Therefore, such research will suffer major technical and quality control issues associated with the harvest of pure CSC populations and the subsequent yield of pure CSCs-EVs components. More efficient methods to isolate pure CSCs-EVs should be developed in the future.

Although the research on CSCs-EVs still has unresolved problems, however, as cancer treatment enters the era of precisely targeted therapy for individuals, we must recognize that the development of targeted therapy for CSCs is an irresistible trend. Therefore, it is necessary to understand the roles of CSCs in the development of cancer, which will undoubtedly be beneficial to the clinical treatment of cancer. We believe that the future is to develop EVs-based CSCs targeted therapy, which is promising to help improve the patient survival.

## References

[1] Bray F (2018). Global cancer statistics 2018: GLOBOCAN estimates of incidence and mortality worldwide for 36 cancers in 185 countries. CA Cancer J. Clin..

[2] Tabassum DP, Polyak K (2015). Tumorigenesis: it takes a village. Nat. Rev. Cancer.

[3] Yuan M (2019). The emerging treatment landscape of targeted therapy in non-small-cell lung cancer. Signal Transduct. Target. Ther..

[4] Dean M, Fojo T, Bates S (2005). Tumour stem cells and drug resistance. Nat. Rev. Cancer.

[5] Dagogo-Jack I, Shaw AT (2018). Tumour heterogeneity and resistance to cancer therapies. Nat. Rev. Clin. Oncol..

[6] Scheele C, Maynard C, van Rheenen J (2016). Intravital insights into heterogeneity, metastasis, and therapy responses. Trends Cancer.

[7] Al-Hajj M (2004). Therapeutic implications of cancer stem cells. Curr. Opin. Genet. Dev..

[8] Shibue T, Weinberg RA (2017). EMT, CSCs, and drug resistance: the mechanistic link and clinical implications. Nat. Rev. Clin. Oncol..

[9] Prieto-Vila M (2017). Drug resistance driven by cancer stem cells and their niche. Int. J. Mol. Sci..

[10] Namee NM, O’Driscoll L (2018). Extracellular vesicles and anti-cancer drug resistance. Biochim. Biophys. Acta Rev. Cancer.

[11] Goel HL, Mercurio AM (2013). VEGF targets the tumour cell. Nat. Rev. Cancer.

[12] Becker A (2016). Extracellular vesicles in cancer: cell-to-cell mediators of metastasis. Cancer Cell..

[13] Sousa D, Lima RT, Vasconcelos MH (2015). Intercellular transfer of cancer drug resistance traits by extracellular vesicles. Trends Mol. Med..

[14] Bach D-H, Hong J-Y, Park HJ, Lee SK (2017). The role of exosomes and miRNAs in drug-resistance of cancer cells. Int. J. Cancer.

[15] Peng D (2018). miR-34c-5p promotes eradication of acute myeloid leukemia stem cells by inducing senescence through selective RAB27B targeting to inhibit exosome shedding. Leukemia.

[16] Conigliaro A (2015). CD90+ liver cancer cells modulate endothelial cell phenotype through the release of exosomes containing H19 lncRNA. Mol. Cancer.

[17] Lapidot T (1994). A cell initiating human acute myeloid leukaemia after transplantation into SCID mice. Nature.

[18] Batlle E, Clevers H (2017). Cancer stem cells revisited. Nat. Med..

[19] Yang L (2020). Targeting cancer stem cell pathways for cancer therapy. Signal Transduct. Target. Ther..

[20] Pützer BM, Solanki M, Herchenröder O (2017). Advances in cancer stem cell targeting: how to strike the evil at its root. Adv. Drug Deliv. Rev..

[21] Clevers H (2011). The cancer stem cell: premises, promises and challenges. Nat. Med..

[22] Shi W, Jin W, Xia L, Yu H (2020). Novel agents targeting leukemia cells and immune microenvironment for prevention and treatment of relapse of acute myeloid leukemia after allogeneic hematopoietic stem cell transplantation. Acta Pharm. Sin. B.

[23] Gasch C, Ffrench B, O’Leary JJ, Gallagher MF (2017). Catching moving targets: cancer stem cell hierarchies, therapy-resistance & considerations for clinical intervention. Mol. Cancer.

[24] Eun K, Ham SW, Kim H (2017). Cancer stem cell heterogeneity: origin and new perspectives on CSC targeting. BMB Rep..

[25] Brower V (2016). Cancer stem cell hypothesis evolves with emerging research. J. Natl Cancer Inst..

[26] Zhou P (2017). The epithelial to – transition (EMT) and cancer stem cells: implication for treatment resistance in pancreatic cancer. Mol. Cancer.

[27] Rossari F, Zucchinetti C, Buda G, Orciuolo E (2020). Tumor dormancy as an alternative step in the development of chemoresistance and metastasis - clinical implications. Cell Oncol. (Dordr.)..

[28] Al-Hajj M (2003). Prospective identification of tumorigenic breast cancer cells. Proc. Natl Acad. Sci. USA.

[29] Eramo A (2008). Identification and expansion of the tumorigenic lung cancer stem cell population. Cell Death Differ..

[30] O’Brien CA, Pollett A, Gallinger S, Dick JE (2007). A human colon cancer cell capable of initiating tumour growth in immunodeficient mice. Nature.

[31] Shimokawa M (2017). Visualization and targeting of LGR5 human colon cancer stem cells. Nature.

[32] Niess H (2015). Side population cells of pancreatic cancer show characteristics of cancer stem cells responsible for resistance and metastasis. Target Oncol..

[33] Kanwal R, Shukla S, Walker E, Gupta S (2018). Acquisition of tumorigenic potential and therapeutic resistance in CD133+ subpopulation of prostate cancer cells exhibiting stem-cell like characteristics. Cancer Lett..

[34] Yan HC (2014). The identification of the biological characteristics of human ovarian cancer stem cells. Eur. Rev. Med. Pharm. Sci..

[35] Singh SK (2003). Identification of a cancer stem cell in human brain tumors. Cancer Res..

[36] Visvader JE, Lindeman GJ (2012). Cancer stem cells: current status and evolving complexities. Cell Stem Cell.

[37] Nguyen PH (2017). Characterization of Biomarkers of tumorigenic and chemoresistant cancer stem cells in human gastric carcinoma. Clin. Cancer Res..

[38] Marjanovic ND, Weinberg RA, Chaffer CL (2013). Cell plasticity and heterogeneity in cancer. Clin. Chem..

[39] Saygin C (2019). Targeting cancer stemness in the clinic: from hype to hope. Cell Stem Cell.

[40] Prasetyanti PR, Medema JP (2017). Intra-tumor heterogeneity from a cancer stem cell perspective. Mol. Cancer.

[41] Tang DG (2012). Understanding cancer stem cell heterogeneity and plasticity. Cell Res..

[42] Zheng H (2018). Single-cell analysis reveals cancer stem cell heterogeneity in hepatocellular carcinoma. Hepatology.

[43] Najafi M, Mortezaee K, Ahadi R (2019). Cancer stem cell (a)symmetry & plasticity: tumorigenesis and therapy relevance. Life Sci..

[44] Wainwright EN, Scaffidi P (2017). Epigenetics and cancer stem cells: unleashing, hijacking, and restricting cellular plasticity. Trends Cancer.

[45] Ciardiello C, Leone A, Budillon A (2018). The crosstalk between cancer stem cells and microenvironment is critical for solid tumor progression: the significant contribution of extracellular vesicles. Stem Cells Int..

[46] Sachs N, Clevers H (2014). Organoid cultures for the analysis of cancer phenotypes. Curr. Opin. Genet. Dev..

[47] Koren E, Fuchs Y (2016). The bad seed: cancer stem cells in tumor development and resistance. Drug Resist. Updat..

[48] Plaks V, Kong N, Werb Z (2015). The cancer stem cell niche: how essential is the niche in regulating stemness of tumor cells?. Cell Stem Cell.

[49] Kim W-T, Ryu CJ (2017). Cancer stem cell surface markers on normal stem cells. BMB Rep..

[50] Wang Z, Zöller M (2019). Exosomes, metastases, and the miracle of cancer stem cell markers. Cancer Metastasis Rev..

[51] Bonnet D, Dick JE (1997). Human acute myeloid leukemia is organized as a hierarchy that originates from a primitive hematopoietic cell. Nat. Med..

[52] Barzegar Behrooz A, Syahir A, Ahmad S (2019). CD133: beyond a cancer stem cell biomarker. J. Drug Target..

[53] Aghajani M (2019). New emerging roles of CD133 in cancer stem cell: signaling pathway and miRNA regulation. J. Cell Physiol..

[54] Xu HS (2017). Cancer stem cell markers in glioblastoma - an update. Eur. Rev. Med. Pharm. Sci..

[55] Erhart F (2019). Gliomasphere marker combinatorics: multidimensional flow cytometry detects CD44+/CD133+/ITGA6+/CD36+ signature. J. Cell Mol. Med..

[56] Rabinovich I (2018). Cancer stem cell markers ALDH1 and CD44+/CD24- phenotype and their prognosis impact in invasive ductal carcinoma. Eur. J. Histochem..

[57] Ortiz RC (2018). CD44 and ALDH1 immunoexpression as prognostic indicators of invasion and metastasis in oral squamous cell carcinoma. J. Oral. Pathol. Med..

[58] de Beça FF (2013). Cancer stem cells markers CD44, CD24 and ALDH1 in breast cancer special histological types. J. Clin. Pathol..

[59] Son MJ (2009). SSEA-1 is an enrichment marker for tumor-initiating cells in human glioblastoma. Cell Stem Cell.

[60] Takashima Y, Kawaguchi A, Yamanaka R (2019). Promising prognosis marker candidates on the status of epithelial-mesenchymal transition and glioma stem cells in glioblastoma. Cells.

[61] Pan T, Xu J, Zhu Y (2017). Self-renewal molecular mechanisms of colorectal cancer stem cells. Int. J. Mol. Med..

[62] Ahmad G, Amiji MM (2017). Cancer stem cell-targeted therapeutics and delivery strategies. Expert Opin. Drug Deliv..

[63] Wu S, Fu L (2018). Tyrosine kinase inhibitors enhanced the efficacy of conventional chemotherapeutic agent in multidrug resistant cancer cells. Mol. Cancer.

[64] Zhao J (2016). Cancer stem cells and chemoresistance: the smartest survives the raid. Pharm. Ther..

[65] Hen O, Barkan D (2020). Dormant disseminated tumor cells and cancer stem/progenitor-like cells: Similarities and opportunities. Semin. Cancer Biol..

[66] Liau BB (2017). Adaptive chromatin remodeling drives glioblastoma stem cell plasticity and drug tolerance. Cell Stem Cell.

[67] Kurtova AV (2015). Blocking PGE2-induced tumour repopulation abrogates bladder cancer chemoresistance. Nature.

[68] Bragado P (2013). TGF-β2 dictates disseminated tumour cell fate in target organs through TGF-β-RIII and p38α/β signalling. Nat. Cell Biol..

[69] Oshimori N, Oristian D, Fuchs E (2015). TGF-β promotes heterogeneity and drug resistance in squamous cell carcinoma. Cell.

[70] Burke AR (2012). The resistance of breast cancer stem cells to conventional hyperthermia and their sensitivity to nanoparticle-mediated photothermal therapy. Biomaterials.

[71] Peitzsch C, Tyutyunnykova A, Pantel K, Dubrovska A (2017). Cancer stem cells: the root of tumor recurrence and metastases. Semin. Cancer Biol..

[72] Theodoropoulos PA (2010). Circulating tumor cells with a putative stem cell phenotype in peripheral blood of patients with breast cancer. Cancer Lett..

[73] Balic M (2006). Most early disseminated cancer cells detected in bone marrow of breast cancer patients have a putative breast cancer stem cell phenotype. Clin. Cancer Res..

[74] Ye X (2015). Distinct EMT programs control normal mammary stem cells and tumour-initiating cells. Nature.

[75] Nandy SB, Lakshmanaswamy R (2017). Cancer stem cells and metastasis. Prog. Mol. Biol. Transl. Sci..

[76] Müller L (2020). Bidirectional crosstalk between cancer stem cells and immune cell subsets. Front. Immunol..

[77] Clara JA, Monge C, Yang Y, Takebe N (2020). Targeting signalling pathways and the immune microenvironment of cancer stem cells - a clinical update. Nat. Rev. Clin. Oncol..

[78] Zhou W (2015). Periostin secreted by glioblastoma stem cells recruits M2 tumour-associated macrophages and promotes malignant growth. Nat. Cell Biol..

[79] Jinushi M (2014). Role of cancer stem cell-associated inflammation in creating pro-inflammatory tumorigenic microenvironments. Oncoimmunology.

[80] Zhang Q, Cai D-J, Li B (2015). Ovarian cancer stem-like cells elicit the polarization of M2 macrophages. Mol. Med. Rep..

[81] Liu L (2017). Anti-CD47 antibody as a targeted therapeutic agent for human lung cancer and cancer stem cells. Front. Immunol..

[82] Theocharides APA (2012). Disruption of SIRPα signaling in macrophages eliminates human acute myeloid leukemia stem cells in xenografts. J. Exp. Med..

[83] Wei J (2010). Glioblastoma cancer-initiating cells inhibit T-cell proliferation and effector responses by the signal transducers and activators of transcription 3 pathway. Mol. Cancer Ther..

[84] Chikamatsu K (2011). Immunoregulatory properties of CD44+ cancer stem-like cells in squamous cell carcinoma of the head and neck. Head. Neck..

[85] Schatton T (2010). Modulation of T-cell activation by malignant melanoma initiating cells. Cancer Res..

[86] Tkach M, Thery C (2016). Communication by extracellular vesicles: where we are and where we need to go. Cell.

[87] Xiao Y (2019). Extracellular vesicles in type 2 diabetes mellitus: key roles in pathogenesis, complications, and therapy. J. Extracell. Vesicles.

[88] Shao H (2018). New technologies for analysis of extracellular vesicles. Chem. Rev..

[89] Thery C, Zitvogel L, Amigorena S (2002). Exosomes: composition, biogenesis and function. Nat. Rev. Immunol..

[90] Zhang J (2015). Exosome and exosomal microRNA: trafficking, sorting, and function. Genomics Proteom. Bioinformatics.

[91] Pegtel DM, Gould SJ (2019). Exosomes. Annu. Rev. Biochem..

[92] van Niel G, D’Angelo G, Raposo G (2018). Shedding light on the cell biology of extracellular vesicles. Nat. Rev. Mol. Cell Biol..

[93] Xu R (2018). Extracellular vesicles in cancer - implications for future improvements in cancer care. Nat. Rev. Clin. Oncol..

[94] Bebelman MP, Smit MJ, Pegtel DM, Baglio SR (2018). Biogenesis and function of extracellular vesicles in cancer. Pharm. Ther..

[95] Christ L (2017). Cellular functions and molecular mechanisms of the ESCRT membrane-scission machinery. Trends Biochem. Sci..

[96] Farooqi AA (2018). Exosome biogenesis, bioactivities and functions as new delivery systems of natural compounds. Biotechnol. Adv..

[97] Ghossoub R (2014). Syntenin-ALIX exosome biogenesis and budding into multivesicular bodies are controlled by ARF6 and PLD2. Nat. Commun..

[98] Kowal J, Tkach M, Thery C (2014). Biogenesis and secretion of exosomes. Curr. Opin. Cell Biol..

[99] Muralidharan-Chari V (2009). ARF6-regulated shedding of tumor cell-derived plasma membrane microvesicles. Curr. Biol..

[100] Kalluri R, LeBleu VS (2020). The biology function and biomedical applications of exosomes. Science.

[101] Akers JC (2013). Biogenesis of extracellular vesicles (EV): exosomes, microvesicles, retrovirus-like vesicles, and apoptotic bodies. J. Neurooncol..

[102] Barile L, Vassalli G (2017). Exosomes: therapy delivery tools and biomarkers of diseases. Pharmacol. Ther..

[103] Huang W (2020). Exosomes with low miR-34c-3p expression promote invasion and migration of non-small cell lung cancer by upregulating integrin α2β1. Signal Transduct. Target. Ther..

[104] Mashouri L (2019). Exosomes: composition, biogenesis, and mechanisms in cancer metastasis and drug resistance. Mol. Cancer.

[105] Kalluri R (2016). The biology and function of exosomes in cancer. J. Clin. Investig..

[106] Zhang L, Yu D (2019). Exosomes in cancer development, metastasis, and immunity. Biochim. Biophys. Acta Rev. Cancer.

[107] Steinbichler TB, Dudás J, Riechelmann H, Skvortsova I-I (2017). The role of exosomes in cancer metastasis. Semin. Cancer Biol..

[108] Wang H, Lu Z, Zhao X (2019). Tumorigenesis, diagnosis, and therapeutic potential of exosomes in liver cancer. J. Hematol. Oncol..

[109] Abels ER, Breakefield XO (2016). Introduction to extracellular vesicles: biogenesis, RNA cargo selection, content, release, and uptake. Cell Mol. Neurobiol..

[110] Chen R (2019). The biological functions and clinical applications of exosomes in lung cancer. Cell Mol. Life Sci..

[111] Kahlert C, Kalluri R (2013). Exosomes in tumor microenvironment influence cancer progression and metastasis. J. Mol. Med..

[112] Wortzel I, Dror S, Kenific CM, Lyden D (2019). Exosome-mediated metastasis: communication from a distance. Dev. Cell..

[113] Xie F (2019). Extracellular vesicles in cancer immune microenvironment and cancer immunotherapy. Adv. Sci. (Weinh.).

[114] Godlewski J (2017). MicroRNA signatures and molecular subtypes of glioblastoma: the role of extracellular transfer. Stem Cell Rep..

[115] Ricklefs F (2016). Extracellular vesicles from high-grade glioma exchange diverse pro-oncogenic signals that maintain intratumoral heterogeneity. Cancer Res..

[116] Han K-Y, Chang J-H, Azar DT (2019). MMP14-containing exosomes cleave VEGFR1 and promote VEGFA-Induced migration and proliferation of vascular endothelial cells. Invest. Ophthalmol. Vis. Sci..

[117] Hsu YL (2017). Hypoxic lung cancer-secreted exosomal miR-23a increased angiogenesis and vascular permeability by targeting prolyl hydroxylase and tight junction protein ZO-1. Oncogene.

[118] Han Y (2019). Exosomes from hypoxia-treated human adipose-derived mesenchymal stem cells enhance angiogenesis through VEGF/VEGF-R. Int. J. Biochem. Cell Biol..

[119] Feng W (2019). Exosomes promote pre-metastatic niche formation in ovarian cancer. Mol. Cancer.

[120] Guo Y (2019). Effects of exosomes on pre-metastatic niche formation in tumors. Mol. Cancer.

[121] Hoshino A (2015). Tumour exosome integrins determine organotropic metastasis. Nature.

[122] Samuel, P., Fabbri, M. & Carter, D. R. F. Mechanisms of drug resistance in cancer: the role of extracellular vesicles. *Proteomics*. **17**, 23–34 (2017). 10.1002/pmic.201600375 28941129

[123] Maacha S (2019). Extracellular vesicles-mediated intercellular communication: roles in the tumor microenvironment and anti-cancer drug resistance. Mol. Cancer.

[124] Latifkar A, Cerione RA, Antonyak MA (2018). Probing the mechanisms of extracellular vesicle biogenesis and function in cancer. Biochem. Soc. Trans..

[125] Levchenko A (2005). Intercellular transfer of P-glycoprotein mediates acquired multidrug resistance in tumor cells. Proc. Natl Acad. Sci. USA.

[126] Goler-Baron V, Sladkevich I, Assaraf YG (2012). Inhibition of the PI3K-Akt signaling pathway disrupts ABCG2-rich extracellular vesicles and overcomes multidrug resistance in breast cancer cells. Biochem. Pharmacol..

[127] Lu JF (2013). Microparticles mediate MRP1 intercellular transfer and the re-templating of intrinsic resistance pathways. Pharm. Res..

[128] Zhou H-l (2013). Intercellular transfer of P-glycoprotein from the drug resistant human bladder cancer cell line BIU-87 does not require cell-to-cell contact. J. Urol..

[129] To KKW (2013). MicroRNA: a prognostic biomarker and a possible druggable target for circumventing multidrug resistance in cancer chemotherapy. J. Biomed. Sci..

[130] Gezer U (2014). Long non-coding RNAs with low expression levels in cells are enriched in secreted exosomes. Cell Biol. Int..

[131] Wei Y (2014). Exosomal miR-221/222 enhances tamoxifen resistance in recipient ER-positive breast cancer cells. Breast Cancer Res. Treat..

[132] Takahashi K (2014). Extracellular vesicle-mediated transfer of long non-coding RNA ROR modulates chemosensitivity in human hepatocellular cancer. FEBS Open Bio.

[133] Takahashi K (2014). Involvement of extracellular vesicle long noncoding RNA (linc-VLDLR) in tumor cell responses to chemotherapy. Mol. Cancer Res..

[134] Li I, Nabet BY (2019). Exosomes in the tumor microenvironment as mediators of cancer therapy resistance. Mol. Cancer.

[135] Bliss SA (2016). Mesenchymal stem cell-derived exosomes stimulate cycling quiescence and early breast cancer dormancy in bone marrow. Cancer Res..

[136] Hu JL (2019). CAFs secreted exosomes promote metastasis and chemotherapy resistance by enhancing cell stemness and epithelial-mesenchymal transition in colorectal cancer. Mol. Cancer.

[137] Ren J (2018). Carcinoma-associated fibroblasts promote the stemness and chemoresistance of colorectal cancer by transferring exosomal lncRNA H19. Theranostics.

[138] Whiteside TL (2016). Exosomes and tumor-mediated immune suppression. J. Clin. Investig..

[139] Graner MW, Schnell S, Olin MR (2018). Tumor-derived exosomes, microRNAs, and cancer immune suppression. Semin. Immunopathol..

[140] Maybruck BT, Pfannenstiel LW, Diaz-Montero M, Gastman BR (2017). Tumor-derived exosomes induce CD8 T cell suppressors. J. Immunother. Cancer.

[141] Chalmin F (2010). Membrane-associated Hsp72 from tumor-derived exosomes mediates STAT3-dependent immunosuppressive function of mouse and human myeloid-derived suppressor cells. J. Clin. Investig..

[142] Plebanek MP (2017). Pre-metastatic cancer exosomes induce immune surveillance by patrolling monocytes at the metastatic niche. Nat. Commun..

[143] Zhou J (2018). Exosomes released from tumor-associated macrophages transfer miRNAs that induce a Treg/Th17 cell imbalance in epithelial ovarian cancer. Cancer Immunol. Res..

[144] Szebeni GJ, Vizler C, Kitajka K, Puskas LG (2017). Inflammation and cancer: extra- and intracellular determinants of tumor-associated macrophages as tumor promoters. Mediators Inflamm..

[145] Sun Z, Wang L, Dong L, Wang X (2018). Emerging role of exosome signalling in maintaining cancer stem cell dynamic equilibrium. J. Cell Mol. Med..

[146] Seo N, Akiyoshi K, Shiku H (2018). Exosome-mediated regulation of tumor immunology. Cancer Sci..

[147] Nakano I, Garnier D, Minata M, Rak J (2015). Extracellular vesicles in the biology of brain tumour stem cells-Implications for inter-cellular communication, therapy and biomarker development. Semin. Cell Dev. Biol..

[148] Sun Z (2020). Glioblastoma stem cell-derived exosomes enhance stemness and tumorigenicity of glioma cells by transferring Notch1 protein. Cell Mol. Neurobiol..

[149] Wang Z (2019). Pancreatic cancer-initiating cell exosome message transfer into noncancer-initiating cells: the importance of CD44v6 in reprogramming. J. Exp. Clin. Cancer Res..

[150] Sharma A (2018). Role of stem cell derived exosomes in tumor biology. Int J. Cancer.

[151] Wang L (2019). CD103-positive CSC exosome promotes EMT of clear cell renal cell carcinoma: role of remote MiR-19b-3p. Mol. Cancer.

[152] Al-Sowayan BS, Al-Shareeda AT, Alrfaei BM (2020). Cancer stem cell-exosomes, unexposed player in tumorigenicity. Front. Pharmacol..

[153] Zhao H, Chen S, Fu Q (2020). Exosomes from CD133 cells carrying circ-ABCC1 mediate cell stemness and metastasis in colorectal cancer. J. Cell Biochem..

[154] Li W (2019). Exosomal FMR1-AS1 facilitates maintaining cancer stem-like cell dynamic equilibrium via TLR7/NFκB/c-Myc signaling in female esophageal carcinoma. Mol. Cancer.

[155] Sun Z (2019). Glioblastoma stem cell-derived exosomes enhance stemness and tumorigenicity of glioma cells by transferring Notch1 protein. Cell Mol. Neurobiol..

[156] Bourkoula E (2014). Glioma-associated stem cells: a novel class of tumor-supporting cells able to predict prognosis of human low-grade gliomas. Stem Cells.

[157] Sun H (2019). The pancreatic cancer-initiating cell marker CD44v6 affects transcription, translation, and signaling: consequences for exosome composition and delivery. J. Oncol..

[158] Wang Z, Zhao K, Hackert T, Zöller M (2018). CD44/CD44v6 a reliable companion in cancer-initiating cell maintenance and tumor progression. Front. Cell Dev. Biol..

[159] Heiler S, Wang Z, Zöller M (2016). Pancreatic cancer stem cell markers and exosomes - the incentive push. World J. Gastroenterol..

[160] Takebe N (2015). Targeting Notch, Hedgehog, and Wnt pathways in cancer stem cells: clinical update. Nat. Rev. Clin. Oncol..

[161] Krause M, Dubrovska A, Linge A, Baumann M (2017). Cancer stem cells: radioresistance, prediction of radiotherapy outcome and specific targets for combined treatments. Adv. Drug Deliv. Rev..

[162] Wang L (2020). Lung CSC-derived exosomal miR-210-3p contributes to a pro-metastatic phenotype in lung cancer by targeting FGFRL1. J. Cell Mol. Med..

[163] Grange C (2011). Microvesicles released from human renal cancer stem cells stimulate angiogenesis and formation of lung premetastatic niche. Cancer Res..

[164] Alzahrani FA (2018). Potential effect of exosomes derived from cancer stem cells and MSCs on progression of DEN-induced HCC in Rats. Stem Cells Int..

[165] Hardin H (2018). Thyroid cancer stem-like cell exosomes: regulation of EMT via transfer of lncRNAs. Lab. Investig..

[166] Weidle UH, Birzele F, Kollmorgen G, Rüger R (2017). Long non-coding RNAs and their role in metastasis. Cancer Genomics Proteom..

[167] Kyuno D (2019). Claudin7-dependent exosome-promoted reprogramming of nonmetastasizing tumor cells. Int. J. Cancer.

[168] Mu W, Wang Z, Zöller M (2019). Ping-pong-tumor and host in pancreatic cancer progression. Front. Oncol..

[169] Schito L, Semenza GL (2016). Hypoxia-inducible factors: master regulators of cancer progression. Trends Cancer.

[170] Yao H, Liu N, Lin MC, Zheng J (2016). Positive feedback loop between cancer stem cells and angiogenesis in hepatocellular carcinoma. Cancer Lett..

[171] Chen X (2020). Epigenetic strategies synergize with PD-L1/PD-1 targeted cancer immunotherapies to enhance antitumor responses. Acta Pharm. Sin. B.

[172] Wang Z-F, Liao F, Wu H, Dai J (2019). Glioma stem cells-derived exosomal miR-26a promotes angiogenesis of microvessel endothelial cells in glioma. J. Exp. Clin. Cancer Res..

[173] Treps L (2017). Glioblastoma stem-like cells secrete the pro-angiogenic VEGF-A factor in extracellular vesicles. J. Extracell. Vesicles.

[174] Sun X (2017). Glioma stem cells-derived exosomes promote the angiogenic ability of endothelial cells through miR-21/VEGF signal. Oncotarget.

[175] Lucero R (2020). Glioma-derived miRNA-containing extracellular vesicles induce angiogenesis by reprogramming brain endothelial cells. Cell Rep..

[176] Skog J (2008). Glioblastoma microvesicles transport RNA and proteins that promote tumour growth and provide diagnostic biomarkers. Nat. Cell Biol..

[177] Spinelli C (2018). Molecular subtypes and differentiation programmes of glioma stem cells as determinants of extracellular vesicle profiles and endothelial cell-stimulating activities. J. Extracell. Vesicles.

[178] Yuan ZX (2016). Targeting strategies for renal cell carcinoma: from renal cancer cells to renal cancer stem cells. Front. Pharmacol..

[179] Lindoso RS, Collino F, Camussi G (2015). Extracellular vesicles derived from renal cancer stem cells induce a pro-tumorigenic phenotype in mesenchymal stromal cells. Oncotarget.

[180] Vera N (2019). Small extracellular vesicles released from ovarian cancer spheroids in response to cisplatin promote the pro-tumorigenic activity of mesenchymal stem cells. Int. J. Mol. Sci..

[181] Yang L (2019). EGFR TKIs impair lysosome-dependent degradation of SQSTM1 to compromise the effectiveness in lung cancer. Signal Transduct. Target. Ther..

[182] Huang L, Fu L (2015). Mechanisms of resistance to EGFR tyrosine kinase inhibitors. Acta Pharm. Sin. B.

[183] Shen M (2019). Chemotherapy-induced extracellular vesicle miRNAs promote breast cancer stemness by targeting ONECUT2. Cancer Res..

[184] Yang Z (2019). Exosomes derived from cancer stem cells of gemcitabine-resistant pancreatic cancer cells enhance drug resistance by delivering miR-210. Cell. Oncol. (Dordr.).

[185] Chen J-H (2019). Ovatodiolide suppresses oral cancer malignancy by down-regulating exosomal Mir-21/STAT3/β-catenin cargo and preventing oncogenic transformation of normal gingival fibroblasts. Cancers.

[186] Kang M, Kim S, Ko J (2019). Roles of CD133 in microvesicle formation and oncoprotein trafficking in colon cancer. FASEB J..

[187] Santos JC (2018). Exosome-mediated breast cancer chemoresistance via miR-155 transfer. Sci. Rep..

[188] Waclaw B (2015). A spatial model predicts that dispersal and cell turnover limit intratumour heterogeneity. Nature.

[189] Hwang W-L (2019). Tumor stem-like cell-derived exosomal RNAs prime neutrophils for facilitating tumorigenesis of colon cancer. J. Hematol. Oncol..

[190] Mirzaei R (2018). Brain tumor-initiating cells export tenascin-C associated with exosomes to suppress T cell activity. Oncoimmunology.

[191] Su C-Y (2020). The biological functions and clinical applications of integrins in cancers. Front. Pharmacol..

[192] Domenis R (2017). Systemic T cells immunosuppression of glioma stem cell-derived exosomes is mediated by monocytic myeloid-derived suppressor cells. PLoS ONE.

[193] Grange C (2015). Role of HLA-G and extracellular vesicles in renal cancer stem cell-induced inhibition of dendritic cell differentiation. BMC Cancer.

[194] Gabrusiewicz K (2018). Glioblastoma stem cell-derived exosomes induce M2 macrophages and PD-L1 expression on human monocytes. Oncoimmunology.

[195] Yang J (2019). Extracellular vesicle lncRNA metastasis-associated lung adenocarcinoma transcript 1 released from glioma stem cells modulates the inflammatory response of microglia after lipopolysaccharide stimulation through regulating miR-129-5p/high mobility group box-1 protein axis. Front. Immunol..

[196] Weissenberger J (2004). IL-6 is required for glioma development in a mouse model. Oncogene.

[197] Brat DJ, Bellail AC, Van Meir EG (2005). The role of interleukin-8 and its receptors in gliomagenesis and tumoral angiogenesis. Neuro-Oncol..

[198] Cheng W-C (2019). RAB27B-activated secretion of stem-like tumor exosomes delivers the biomarker microRNA-146a-5p, which promotes tumorigenesis and associates with an immunosuppressive tumor microenvironment in colorectal cancer. Int. J. Cancer.

[199] Thuma F, Zöller M (2014). Outsmart tumor exosomes to steal the cancer initiating cell its niche. Semin. Cancer Biol..

[200] Lv L-L (2020). Exosomal miRNA-19b-3p of tubular epithelial cells promotes M1 macrophage activation in kidney injury. Cell Death Differ..

[201] Lin X-J (2018). Hepatocellular carcinoma cell-secreted exosomal microRNA-210 promotes angiogenesis in vitro and in vivo. Mol. Ther. Nucleic Acids.

[202] Zhang R (2018). Long non-coding RNA Linc-ROR is upregulated in papillary thyroid carcinoma. Endocr. Pathol..

[203] Teeuwssen M, Fodde R (2019). Wnt signaling in ovarian cancer stemness, EMT, and therapy resistance. J. Clin. Med..

[204] Huang T-X, Guan X-Y, Fu L (2019). Therapeutic targeting of the crosstalk between cancer-associated fibroblasts and cancer stem cells. Am. J. Cancer Res..

[205] Chan T-S, Shaked Y, Tsai KK (2019). Targeting the interplay between cancer fibroblasts, mesenchymal stem cells, and cancer stem cells in desmoplastic cancers. Front. Oncol..

[206] Najafi M, Farhood B, Mortezaee K (2019). Cancer stem cells (CSCs) in cancer progression and therapy. J. Cell Physiol..

[207] Shen M (2019). Chemotherapy-induced extracellular vesicle miRNAs promote breast cancer stemness by targeting. Cancer Res..

[208] Li Z (2018). Tumor-derived exosomal lnc-Sox2ot promotes EMT and stemness by acting as a ceRNA in pancreatic ductal adenocarcinoma. Oncogene.

[209] Kuc N (2018). Pancreatic ductal adenocarcinoma cell secreted extracellular vesicles containing ceramide-1-phosphate promote pancreatic cancer stem cell motility. Biochem. Pharmacol..

[210] Ramteke A (2015). Exosomes secreted under hypoxia enhance invasiveness and stemness of prostate cancer cells by targeting adherens junction molecules. Mol. Carcinog..

[211] Liang SB, Fu LW (2017). Application of single-cell technology in cancer research. Biotechnol. Adv..

[212] Annett S, Robson T (2018). Targeting cancer stem cells in the clinic: current status and perspectives. Pharmacol. Ther..

[213] Lytle NK, Barber AG, Reya T (2018). Stem cell fate in cancer growth, progression and therapy resistance. Nat. Rev. Cancer.

[214] Wang J, Zheng Y, Zhao M (2016). Exosome-based cancer therapy: implication for targeting cancer stem cells. Front. Pharmacol..

[215] Ha D, Yang N, Nadithe V (2016). Exosomes as therapeutic drug carriers and delivery vehicles across biological membranes: current perspectives and future challenges. Acta Pharm. Sin. B.

[216] Liao W (2019). Exosomes: The next generation of endogenous nanomaterials for advanced drug delivery and therapy. Acta Biomater..

[217] Kibria G (2018). Exosomes as a drug delivery system in cancer therapy: potential and challenges. Mol. Pharm..

[218] Steinbichler TB (2019). Therapy resistance mediated by exosomes. Mol. Cancer.

[219] Yong T (2019). Tumor exosome-based nanoparticles are efficient drug carriers for chemotherapy. Nat. Commun..

[220] Zhao L (2020). Exosome-mediated siRNA delivery to suppress postoperative breast cancer metastasis. J. Control Release.

[221] Arabi L, Badiee A, Mosaffa F, Jaafari MR (2015). Targeting CD44 expressing cancer cells with anti-CD44 monoclonal antibody improves cellular uptake and antitumor efficacy of liposomal doxorubicin. J. Control Release.

[222] Tian Y (2014). A doxorubicin delivery platform using engineered natural membrane vesicle exosomes for targeted tumor therapy. Biomaterials.

[223] Qi H (2016). Blood exosomes endowed with magnetic and targeting properties for cancer therapy. ACS Nano..

[224] Allahverdiyev AM (2018). Current aspects in treatment of breast cancer based of nanodrug delivery systems and future prospects. Artif. Cells Nanomed. Biotechnol..

[225] Greco KA (2016). PLK-1 silencing in bladder cancer by siRNA delivered with exosomes. Urology.

[226] Lou G (2015). Exosomes derived from miR-122-modified adipose tissue-derived MSCs increase chemosensitivity of hepatocellular carcinoma. J. Hematol. Oncol..

[227] Cao S (2018). FOXC1 induces cancer stem cell-like properties through upregulation of beta-catenin in NSCLC. J. Exp. Clin. Cancer Res..

[228] Bai X (2018). Cancer stem cell in breast cancer therapeutic resistance. Cancer Treat. Rev..

[229] De Robertis M, Poeta ML, Signori E, Fazio VM (2018). Current understanding and clinical utility of miRNAs regulation of colon cancer stem cells. Semin. Cancer Biol..

[230] Sun J-H, Luo Q, Liu L-L, Song G-B (2016). Liver cancer stem cell markers: progression and therapeutic implications. World J. Gastroenterol..

[231] Zeijlemaker W (2019). CD34CD38 leukemic stem cell frequency to predict outcome in acute myeloid leukemia. Leukemia.

[232] Brungs D (2016). Gastric cancer stem cells: evidence, potential markers, and clinical implications. J. Gastroenterol..

[233] Lukenda A (2016). Expression and prognostic value of putative cancer stem cell markers CD117 and CD15 in choroidal and ciliary body melanoma. J. Clin. Pathol..

[234] Liu S (2013). Expression of intercellular adhesion molecule 1 by hepatocellular carcinoma stem cells and circulating tumor cells. Gastroenterology.

[235] Wahab SMR, Islam F, Gopalan V, Lam AK-Y (2017). The identifications and clinical implications of cancer stem cells in colorectal cancer. Clin. Colorectal Cancer.

[236] Tachezy M (2014). Activated leukocyte cell adhesion molecule (CD166): an “inert” cancer stem cell marker for non-small cell lung cancer?. Stem Cells.

[237] Djirackor L, Kalirai H, Coupland SE, Petrovski G (2019). CD166high uveal melanoma cells represent a subpopulation with enhanced migratory capacity. Investig. Ophthalmol. Vis. Sci..

[238] Sakabe T (2017). CD117 expression is a predictive marker for poor prognosis in patients with non-small cell lung cancer. Oncol. Lett..

[239] Cole, J. M., Joseph, S., Sudhahar, C. G. & Cowden Dahl, K. D. Enrichment for chemoresistant ovarian cancer stem cells from human cell lines. *J. Vis. Exp.* **91**, 51891 (2014). 10.3791/51891 PMC4828064 25285606

[240] Pearson AT, Jackson TL, Nör JE (2016). Modeling head and neck cancer stem cell-mediated tumorigenesis. Cell Mol. Life Sci..

[241] Louphrasitthiphol P, Chauhan J, Goding CR (2020). ABCB5 is activated by MITF and β-catenin and is associated with melanoma differentiation. Pigment Cell Melanoma Res..

[242] Skvortsov S, Skvortsova I-I, Tang DG, Dubrovska A (2018). Concise review: prostate cancer stem cells: current understanding. Stem Cells.

[243] Aghaalikhani N (2019). Cancer stem cells as a therapeutic target in bladder cancer. J. Cell Physiol..

[244] Brooks DLP (2016). ITGA6 is directly regulated by hypoxia-inducible factors and enriches for cancer stem cell activity and invasion in metastatic breast cancer models. Mol. Cancer.

[245] Colombel M (2012). Increased expression of putative cancer stem cell markers in primary prostate cancer is associated with progression of bone metastases. Prostate.

[246] Rasti A (2017). Reduced expression of CXCR4, a novel renal cancer stem cell marker, is associated with high-grade renal cell carcinoma. J. Cancer Res. Clin. Oncol..

[247] Osman WM, Shash LS, Ahmed NS (2017). Emerging role of nestin as an angiogenesis and cancer stem cell marker in epithelial ovarian cancer: immunohistochemical study. Appl. Immunohistochem. Mol. Morphol..

[248] Neradil J, Veselska R (2015). Nestin as a marker of cancer stem cells. Cancer Sci..

[249] Costa CD (2019). Characterization of OCT3/4, Nestin, NANOG, CD44 and CD24 as stem cell markers in canine prostate cancer. Int. J. Biochem. Cell Biol..

